# Physiological changes in captive elephants in Northern Thailand as a result of the COVID-19 tourism ban – muscle, liver, metabolic function, and body condition

**DOI:** 10.3389/fvets.2023.1303537

**Published:** 2023-12-21

**Authors:** Jarawee Supanta, Janine L. Brown, Pakkanut Bansiddhi, Chatchote Thitaram, Veerasak Punyapornwithaya, Khanittha Punturee, Nopphamas Somboon, Patcharapa Towiboon, Jaruwan Khonmee

**Affiliations:** ^1^Faculty of Veterinary Medicine, Chiang Mai University, Chiang Mai, Thailand; ^2^Center of Elephant and Wildlife Health, Chiang Mai University Animal Hospital, Chiang Mai, Thailand; ^3^Elephant, Wildlife, and Companion Animals Research Group, Chiang Mai University, Chiang Mai, Thailand; ^4^Center for Species Survival, Smithsonian Conservation Biology Institute, Front Royal, VA, United States; ^5^Department of Medical Technology, Faculty of Associated Medical Sciences, Chiang Mai University, Chiang Mai, Thailand; ^6^Small Animal Hospital, Faculty of Veterinary Medicine, Chiang Mai University, Chiang Mai, Thailand

**Keywords:** elephant, health, tourism, COVID-19, Thailand

## Abstract

The international travel ban initiated in March 2020 due to the COVID-19 pandemic greatly affected how captive elephants were managed in Thailand. A lack of tourists and associated income meant elephants were chained longer with reduced food provisions, had fewer mahouts, and limited exercise like riding, which likely affected health and welfare. Fifty-eight elephants from six tourist camps were assigned a body condition score (BCS) and blood samples were collected monthly for 2 years during the travel ban to measure: (1) muscle enzymes [creatine kinase (CK), aspartate aminotransferase (AST)]; (2) liver enzymes [aspartate aminotransferase (AST), alkaline phosphatase (ALP), gamma-glutamyl transferase (GGT)]; (3) lipids [total cholesterol (TC), triglycerides (TG), low (LDL) and high (HDL) density lipoproteins]; and metabolic function [glucose, insulin, fructosamine]. Serum CK concentrations were lower at the end of the study, possibly due to no tourist activities like riding. Changes in liver function included increased AST and ALP, also possibly due to physical inactivity. Feeding less bananas and sugar cane was associated with fewer elephants in the obese category and lower TG concentrations. However, increases in glucose, insulin and fructosamine were observed as local people returned to feed elephants after lifting travel restrictions. In sum, changes in several health biomarkers were observed in association with restricted activities and food resources. Camps need better plans to meet the health and welfare needs of elephants during any future disruptions to the tourism industry, although reduced feeding of sweet treats appeared to have positive effects on body condition and metabolic function.

## Introduction

Asian elephants (*Elephas maximus*) are the national symbol of Thailand and listed as endangered on the IUCN Red List since 1986. Elephants are an integral part of Thai and Buddhist culture, as well as being important to the country’s economy through elephant-based tourism. Today, there are ~3,500 captive elephants in Thailand, most (95%) privately owned (1) in tourist camps. The majority are in the north and northeast of the country (~60%), primarily in Chiang Mai province. A survey of elephant camps in the region found tourist activities primarily consisted of saddle riding, bareback riding, elephant show, bathing, feeding, and observation only (2). All of these activities could have varying impacts on the health and welfare of working elephants, and so have been the focus of studies to identify evidence-based best practices for elephant management (2–10). Negative outcomes of elephant tourism include wounds from improper uses of restraint equipment (ankus or chains) (10), increased adrenal glucocorticoid hormones during the high tourist season (3–5), and high rates of stereotypic behaviors (11). In previous studies, half of the elephants were overweight or obese based on body condition scores (BCS), although adequate exercise (including riding) and limiting high calorie treats (bananas and sugar cane) were associated with better body condition, metabolic function and lipid profiles (3, 5, 9). Rigorous and enforceable elephant welfare standards in Thailand are lacking, but increased scrutiny by animal rights groups has led to more tourists having an awareness of animal welfare issues. In 2015, Asian Captive Elephant Standards, an independent organization based in Thailand, was created to audit and certify elephant venues using guidelines based in part on these research findings, resulting in notable improvements. But then the global pandemic hit, and the tourism landscape changed significantly (12).

Upon recognition in March 2020 of SARS-CoV-2, the virus that causes COVID-19, the Thai government banned all international travel (13), severely reducing foreign tourism income and resulting in the closure of all elephant camps. Reductions in exercise opportunities and food provisions, increases in chaining time, and fewer mahouts caring for elephants all were observed (12), raising concerns about impacts on health and welfare. Extensive baseline data from studies of this population before the pandemic (3–6, 8, 10) provided a unique opportunity to examine how COVID-19 induced management changes directly affected elephant physiology. Thus, this study continued assessment of body condition and collection of blood samples during the 2-year travel ban for measurements of biomarkers associated with (1) muscle enzymes [creatine kinase (CK) and aspartate aminotransferase (AST)], (2) liver enzymes [aspartate aminotransferase (AST), alkaline phosphatase (ALP) and gamma-glutamyl transferase (GGT)], (3) lipid profiles [total cholesterol (TC), triglyceride (TG), low density lipoprotein (LDL) and high density lipoprotein (HDL)], and metabolic functions [plasma glucose, serum insulin, glucose:insulin (G:I) ratio, and serum fructosamine]. The goal was to understand the impact of a global pandemic and its downstream effects on the health and welfare of an iconic, culturally and economically important species to Thailand. This information is needed to make science-based recommendations, and identify areas that camps need to improve in order to meet basic elephant needs during crises like the COVID-19 pandemic.

## Materials and methods

### Human and animal ethics statements

This study was approved by the Human Research Ethics Committee (HS1/2564) and the Institutional Animal Care and Use Committee, Faculty of Veterinary Medicine (FVM), Chiang Mai University (CMU), Chiang Mai, Thailand (FVM-ACUC; S4/2564).

### Study population

A total of 58 healthy elephants from six elephant camps in the Chiang Mai area were studied (Supplementary Table 1), including 14 males (32.9 ± 3.3 years of age, range, 19–56 years) and 44 females (38.8 ± 1.4 years of age, range, 20–56 years) as of April 2020. Two camps were considered small (<10 elephants), one was medium (10–30 elephants), and three were large (>30 elephants) size. Before the countrywide lockdown, elephants were involved in riding with a saddle (Camp B, C, E, and F) or bareback (Camp B), elephant shows (Camp B and C), and tourist feeding (all camps). After the lockdown, all tourist activities ceased (Supplementary Table 1) (12).

### Data collection

**Figure 1:**
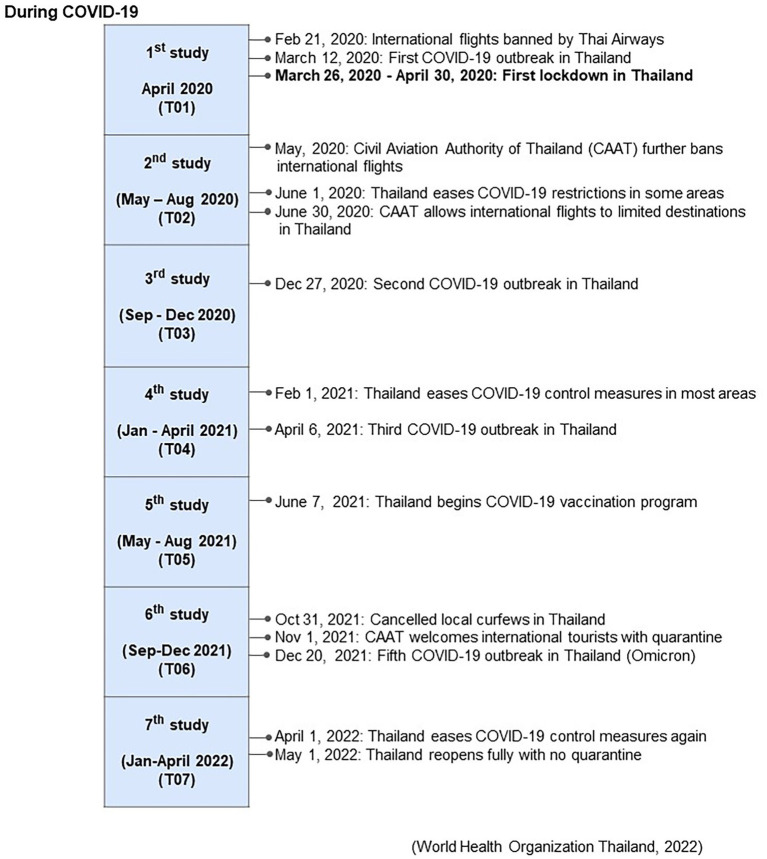
Associated events during the COVID-19 pandemic beginning in April 2020 (T01), and across seven periods of management surveys and blood sample collections (T02-T07) during the 2 years of the study from Supanta et al. (12).

Data collection commenced in April 2020, a month after the countrywide lockdown, and continued through April 2022. The study consisted of camp questionnaire interviews, collection of blood samples to assess muscle, liver, and metabolic function, and body condition scoring. Data were collected monthly for the first year (April 2020 to April 2021), and then every 4 months for the second year (May 2021 to April 2022), resulting in 16 collections that were then divided into seven time periods (T01-T07) (Figure 1). Pre COVID-19 data (T00) obtained from Supanta et al. (12) were included in some comparisons.

### Camp management questionnaire

As described in Supanta et al. (12), interviews were conducted with camp owners, managers, and/or veterinarians to gather information on numbers of tourists, elephants and mahouts (elephant keepers), tourist activities (riding, walking, shows, feeding), chaining duration and length, and provisioning of forage and supplements. Questions about camp management before COVID-19 were included in the first survey to capture data on operations in 2019 before the international travel ban in Thailand (Supplementary Table 1) (12).

### Sample collection

At each collection time, blood (~9 mL) was collected from an ear vein and placed into two tubes: serum separator tubes (SST) (~7 mL) and sodium fluoride tubes (NaF) (~2 mL). SST tubes were used for analysis of muscle enzymes (CK and AST), liver enzymes (AST, ALP, and GGT), metabolic markers including lipid profiles (TC, TG, LDL, and HDL), and metabolic functions (serum fructosamine, serum insulin and G:I ratio). NaF tubes were used for plasma glucose measurements. Blood in SST tubes were centrifuged (Hettich, Westphalia, Germany) at 1200 x g for 15 min, while NaF tubes were centrifuged at 865 x g for 10 min. Serum and plasma samples were stored at −20°C until processing and analysis.

### Muscle and liver enzymes

Serum CK was measured by a DGKC IFCC 37°C method, AST was measured by a pyridoxal-5-phosphate FS (P-5-P) method, ALP was measured by a kinetic photometric method, and GGT was measured by a Szasz mod. /IFCC stand method. All were quantified using an Automated Clinical Chemistry Analyzer (Sysmex; BX-3010, Sysmex Corporation of Japan). Assay sensitivity was 3 U/L (99.9% confidence) for CK, 2 U/L for AST (99.9% confidence), 0.5 U/L for ALP (99.9% confidence), and 0.3 U/L for GGT (99.9% confidence).

### Lipid profiles

Serum lipids were quantified using an Automated Clinical Chemistry Analyzer (Sysmex; BX-3010, Sysmex Corporation of Japan). TC was measured by a cholesterol oxidase-peroxidase (CHOD-PAP) method. TGs were measured by a colorimetric enzymatic test using glycerol-3-phosphate-oxidase (GPO) method, LDL-C and HDL were measured using homogeneous assay methods, with a sensitivity of 0.5 mg/dL (99.9% confidence). The lowest measurable concentration was 2 mg/dL (99.7% confidence) for TG, and 1 mg/dL for both LDL and HDL (99.7% confidence).

### Plasma glucose, serum insulin, G:I, and serum fructosamine

Plasma glucose was measured by a hexokinase method using an automated glucose analyzer (Glucinet T01-149, Bayer, Barcelona, Spain), with quinoneimine measured at 530 nm. Serum insulin concentrations were measured by a solid-phase two-site bovine insulin enzyme immunoassay (EIA; CAT. No. 10-1,113-01; Mercodia, Uppsala, Sweden), validated for elephants (14). Colorimetric responses were determined by a spectrophotometer at 450 nm filter with an Opsys MR Microplate Reader (TECAN Sunrise™ microplate reader; Salzburg, Austria). Serum fructosamine was measured by a colorimetric method using nitrobluetetrazolium (15) in a Biosystems BA400 clinical chemistry analyzer (Biosystems S.A., Barcelona, Spain). All samples were analyzed in duplicate; intra- and inter-assay CV’s were < 10 and < 15%, respectively.

### Body condition scoring

Body condition was scored (1 = thinnest; 5 = fattest) at the time of sample collection based on rear and side view photographs that permitted visual evaluation of the back, rib and pelvic bone areas (14) adapted for elephants in Thailand (5). All photos were evaluated by three experienced elephant veterinarians working with captive elephants at CMU, and the scores averaged.

### Statistical analyses

Statistical analyses were performed using R version 4.3.1 (16). Descriptive data from the questionnaires and biomarker concentrations are presented as a mean ± standard error of the mean (SEM) and the range. Median, mean and percentage data were calculated for body condition. Repeated measures data were analyzed using Generalized Estimating Equation (GEE) in R (17), package geepack, function geeglm, under the optimal autoregressive order 1 correlation structure (18). Univariate analysis was conducted and any variable with *p* < 0.15 was included in the multivariate analysis. Tukey’s multiple comparisons of least-squares mean (LS-mean) were used as *post hoc* tests to determine differences between variable categories. Pearson correlation tests were used to determine associations between continuous variables in the univariate model. Sex and Age were included in multivariate model as covariate factors. For the GEE analysis of management factors, the final model was selected based on the smallest quasi-likelihood under the independence model criterion (QIC) values (18) using package MuMIn, function dredge (19). Reference categories were chosen following a previous study (8, 10) including Sex = male; Time = T01 (Apr 2020, the beginning of the travel ban); Camp = F (smallest camp). Seasonal (summer, 16 February–15 May; rainy, 16 May–15 October; winter, 16 October–15 February) (20, 21) differences were analyzed by Tukey’s *post-hoc* tests after GEE analyses. The significance level was set at *α* = 0.05.

## Results

### Descriptive statistics of camp management variables

Data (*n* = 420 observations) were obtained on 58 elephants. Camp differences in management practices before the COVID-19 travel ban (T00) compared to the start of the lockdown (T01) and each year subsequent (T04, T07) are shown in Supplementary Table 1, with camp differences for muscle, liver, metabolic markers and BCS in Supplementary Table 2. Univariate and multivariate GEE results are presented in Supplementary Tables 3–15. Table 1 summarizes the results of multivariate GEE analyses of category and continuous camp management variables associated with muscle and liver enzymes, lipid profiles, metabolic function and BCS. LS-mean analyses are provided in Table 2 and seasonal effects given in Supplementary Table 16. All camps experienced reductions in tourist numbers that had begun to recover by the end of the study, although only two had notable increases (Camp E, 20%; Camp F, 47%) with the rest increasing by <5%. Mahout numbers were reduced by 33–70% in all camps. Overall elephant numbers were stable in one camp (Camp F), but decreased by nearly half or more in the others, with corresponding declines in participating elephants, although generally not until a year after the lockdown. All elephants were chained for 16 h/day or more even before the travel ban, and except in one camp (Camp E) chaining time was decreased; two camps (Camp B and D) chained for 48 consecutive hours in T04. Two camps (Camp E and F) (25.6%) also reduced chain lengths compared to pre-COVID-19. In one camp (Camp E), chain length was decreased from 5 m to only 1.2 m, although chain time was the lowest and actually decreased from 17 to 14.6 h/day. Subsequently, walking distance was reduced by three-quarters or more in T04 or T07 at all venues except Camp F that maintained a 3 km/day regimen. There was one camp (Camp B) that increased the average amount of roughage provided from 120 kg/day to 131.8 kg/day, while three others (Camp A, C, and F) offered reduced forage as the study progressed. All camps fed fewer high-energy supplements. Three began feeding more at the end of the study, although not to pre-pandemic levels (Supplementary Table 1).

**Table 1:** Summary of significant relationships from multivariate GEE analyses of category and continuous camp management variables associated with muscle and liver enzymes, lipid profiles, metabolic function, and body condition scores (BCS).

Variables	Category camp management variables			Continuous camp management variables						Parameters
Sex	Time	Camp	Age	Walking distance (km/day)	Chain length (m)	Chain hour (h/day)	Roughage (kg/day)	Supplement (kg/day)
**Muscle and liver enzymes**									
Creatine kinase (U/L)	NS	S	S	NS	NS	NS	S (+)	NS	NS
Aspartate aminotransferase (U/L)	NS	S	S	NS	NS	S (+)	NS	NS	NS
Alkaline phosphatase (U/L)	NS	S	S	S (+)	NS	NS	NS	NS	NS
Gamma-glutamyl transferase (U/L)	NS	NS	S	S (+)	NS	NS	NS	NS	NS
**Lipids**									
Total cholesterol (mg/dl)	S	S	S	NS	NS	NS	NS	S (+)	NS
Triglyceride (mg/dl)	NS	S	S	S (−)	S (−)	NS	NS	S (+)	S (−)
Low density lipoprotein (mg/dl)	S	S	S	NS	NS	NS	NS	NS	NS
High density lipoprotein (mg/dl)	NS	S	NS	NS	NS	S (+)	S (−)	NS	NS
**Metabolic function**									
Plasma glucose (mg/dl)	NS	S	S	NS	S (+)	NS	NS	NS	NS
Serum insulin (μg/l)	NS	S	S	NS	S (+)	NS	NS	NS	NS
Glucose to insulin ratio	NS	S	S	NS	NS	NS	NS	NS	NS
Serum fructosamine (mM)	S	S	NS	NS	S (−)	NS	NS	NS	NS
Body condition score (1–5)	S	S	S	NS	NS	NS	NS	NS	S (+)

NS, no significance; S, significance.

Parenthesis: + = positive relationship; − = negative relationship on muscle and liver enzymes, lipid profiles, metabolic functions and BCS.

**Table 2:** LS-mean (± SEM) muscle and liver enzymes, lipid profiles, metabolic function and body condition scores (BCS) of Asian elephants (*n* = 58) in relation to categorical demographic and management variables from the final GEE models.

Variable		*N*	CK (U/L)	AST (U/L)	ALP (U/L)	GGT (U/L)	TC (mg/dl)	TG (mg/dl)	LDL (mg/dl)	HDL (mg/dl)	Glucose (mg/dl)	Insulin (μg/l)	G:I	Fructosamine (mM)	BCS (1–5)
Sex															
	Male	14	311.0 ± 18.8^a^	22.9 ± 0.7^b^	51.9 ± 2.6^b^	5.3 ± 0.3^a^	51.1 ± 1.4^b^	24.1 ± 1.3^a^	33.7 ± 1.0^b^	13.3 ± 0.5^a^	91.3 ± 2.8^a^	0.65 ± 0.10^a^	299.0 ± 31.8^a^	1.44 ± 0.01^b^	3.2 ± 0.1^a^
	Female	44	320.0 ± 8.7^a^	20.6 ± 0.3^a^	48.6 ± 1.1^a^	5.0 ± 0.1^a^	43.1 ± 0.6^a^	22.7 ± 0.7^a^	27.8 ± 0.5^a^	11.5 ± 0.2^a^	90.8 ± 1.3^a^	0.70 ± 0.04^b^	250.0 ± 9.9^a^	1.42 ± 0.01^a^	3.6 ± 0.1^b^
Time															
	T01	58	354.6 ± 17.1^b^	19.5 ± 0.6^a^	41.0 ± 1.8^a^	5.3 ± 0.4^a^	47.1 ± 1.3^c^	33.3 ± 1.6^d^	31.8 ± 1.0^b^	14.5 ± 0.6^d^	87.3 ± 1.9^ab^	0.49 ± 0.10^ab^	296.0 ± 31.3^ab^	1.38 ± 0.02^a^	3.9 ± 0.1^c^
	T02	54	310.3 ± 11.9^b^	19.8 ± 0.5^a^	39.7 ± 1.8^a^	5.3 ± 0.3^a^	46.9 ± 1.2^c^	30.1 ± 1.2^d^	31.6 ± 0.8^b^	11.8 ± 0.3^bc^	92.8 ± 1.7^bc^	0.42 ± 0.06^a^	360.0 ± 18.5^b^	1.39 ± 0.01^a^	3.7 ± 0.1^c^
	T03	51	367.5 ± 19.3^b^	21.6 ± 0.5^ab^	44.4 ± 1.8^ab^	5.3 ± 0.2^a^	48.6 ± 1.3^c^	23.4 ± 0.9^c^	33.5 ± 0.1^b^	13.1 ± 0.4^cd^	80.9 ± 1.8^a^	0.71 ± 0.08^b^	278.0 ± 18.9^a^	1.40 ± 0.01^ab^	3.7 ± 0.1^c^
	T04	46	237.8 ± 9.1^a^	20.7 ± 0.6^ab^	45.6 ± 2.1^ab^	4.5 ± 0.2^a^	41.3 ± 1.0^ab^	19.8 ± 0.9^b^	26.1 ± 0.7^a^	11.0 ± 0.3^ab^	87.3 ± 2.3^ab^	0.70 ± 0.09^ab^	270.0 ± 17.8^a^	1.38 ± 0.01^a^	3.3 ± 0.1^b^
	T05	40	381.8 ± 27.5^b^	24.0 ± 1.0^b^	51.0 ± 2.8^bc^	5.2 ± 0.3^a^	44.3 ± 1.6^bc^	19.5 ± 1.6^abc^	26.9 ± 1.1^a^	10.4 ± 0.4^ab^	91.7 ± 3.7^abc^	0.73 ± 0.13^ab^	238.0 ± 24.5^a^	1.46 ± 0.01^ab^	2.8 ± 0.1^a^
	T06	38	379.0 ± 36.5^b^	22.1 ± 0.9^ab^	61.3 ± 3.0^cd^	5.2 ± 0.3^a^	43.2 ± 1.5^abc^	15.0 ± 1.2^a^	27.0 ± 1.1^a^	10.5 ± 0.4^ab^	80.3 ± 4.3^ab^	0.69 ± 0.11^ab^	198.0 ± 23.6^a^	1.51 ± 0.02^b^	2.7 ± 0.1^a^
	T07	36	196.7 ± 18.9^a^	23.4 ± 1.0^b^	64.1 ± 3.1^d^	4.5 ± 0.3^a^	38.0 ± 1.3^a^	14.8 ± 0.0^a^	22.8 ± 0.9^a^	10.3 ± 0.4^a^	104.1 ± 4.0^c^	0.69 ± 0.14^ab^	233.0 ± 31.4^a^	1.47 ± 0.02^ab^	2.8 ± 0.1^a^
Camp															
	A	14	300.0 ± 13.4^ab^	23.5 ± 1.0^b^	47.9 ± 2.0^b^	4.9 ± 0.2^ab^	50.9 ± 2.0^c^	22.4 ± 0.8^b^	33.4 ± 0.8^c^	12.6 ± 0.4^a^	104.5 ± 2.5^c^	1.13 ± 0.10^b^	191.0 ± 16.8^a^	1.43 ± 0.01^a^	3.4 ± 0.1^b^
	B	14	364.0 ± 14.2^c^	19.5 ± 0.5^a^	56.2 ± 1.9^c^	5.0 ± 0.2^ab^	37.8 ± 0.8^a^	22.4 ± 1.2^b^	23.2 ± 0.6^a^	11.9 ± 0.5^a^	87.1 ± 1.5^ab^	0.71 ± 0.11^a^	242.0 ± 19.0^ab^	1.43 ± 0.01^a^	3.9 ± 0.1^c^
	C	12	307.0 ± 15.8^abc^	21.3 ± 0.7^ab^	51.2 ± 1.8^bc^	4.7 ± 0.3^a^	44.7 ± 0.9^b^	17.9 ± 0.9^a^	28.6 ± 0.7^b^	11.7 ± 0.2^a^	81.4 ± 2.0^a^	0.38 ± 0.06^a^	329.0 ± 23.3^c^	1.42 ± 0.01^a^	3.1 ± 0.1^b^
	D	10	360.0 ± 18.8^bc^	20.9 ± 0.8^ab^	54.6 ± 2.2^bc^	5.9 ± 0.3^b^	36.6 ± 1.1^a^	22.5 ± 1.0^b^	23.1 ± 0.9^a^	10.9 ± 0.4^a^	86.5 ± 1.6^ab^	0.59 ± 0.08^a^	258.0 ± 20.8^abc^	1.43 ± 0.01^a^	3.8 ± 0.1^c^
	E	5	240.0 ± 17.7^a^	19.1 ± 0.9^a^	52.3 ± 2.2^bc^	4.8 ± 0.2^a^	48.2 ± 1.8^bc^	23.8 ± 1.8^ab^	32.3 ± 1.3^bc^	11.8 ± 0.3^a^	94.4 ± 3.8^bc^	0.71 ± 0.11^a^	246.0 ± 22.6^abc^	1.46 ± 0.02^a^	3.3 ± 0.1^b^
	F	3	254.0 ± 15.6^a^	23.7 ± 1.0^b^	35.3 ± 2.0^a^	5.3 ± 0.2^ab^	47.0 ± 1.6^bc^	24.6 ± 2.0^b^	30.6 ± 1.0^bc^	11.6 ± 0.7^a^	81.2 ± 4.5^ab^	0.37 ± 0.10^a^	340.0 ± 32.6^bc^	1.40 ± 0.02^a^	2.0 ± 0.2^a^

^a,b,c^Different letters within columns indicate significant differences for each variable (*p* < 0.001).

Creatine kinase (CK), aspartate aminotransferase (AST), alkaline phosphatase (ALP), gamma-glutamyl transferase (GGT), total cholesterol (TC), triglyceride (TG), high density lipoprotein (HDL), low density lipoprotein (LDL), glucose to insulin ratio (G:I), body condition score (BCS).

### Muscle and liver enzymes

**Figure 2:**
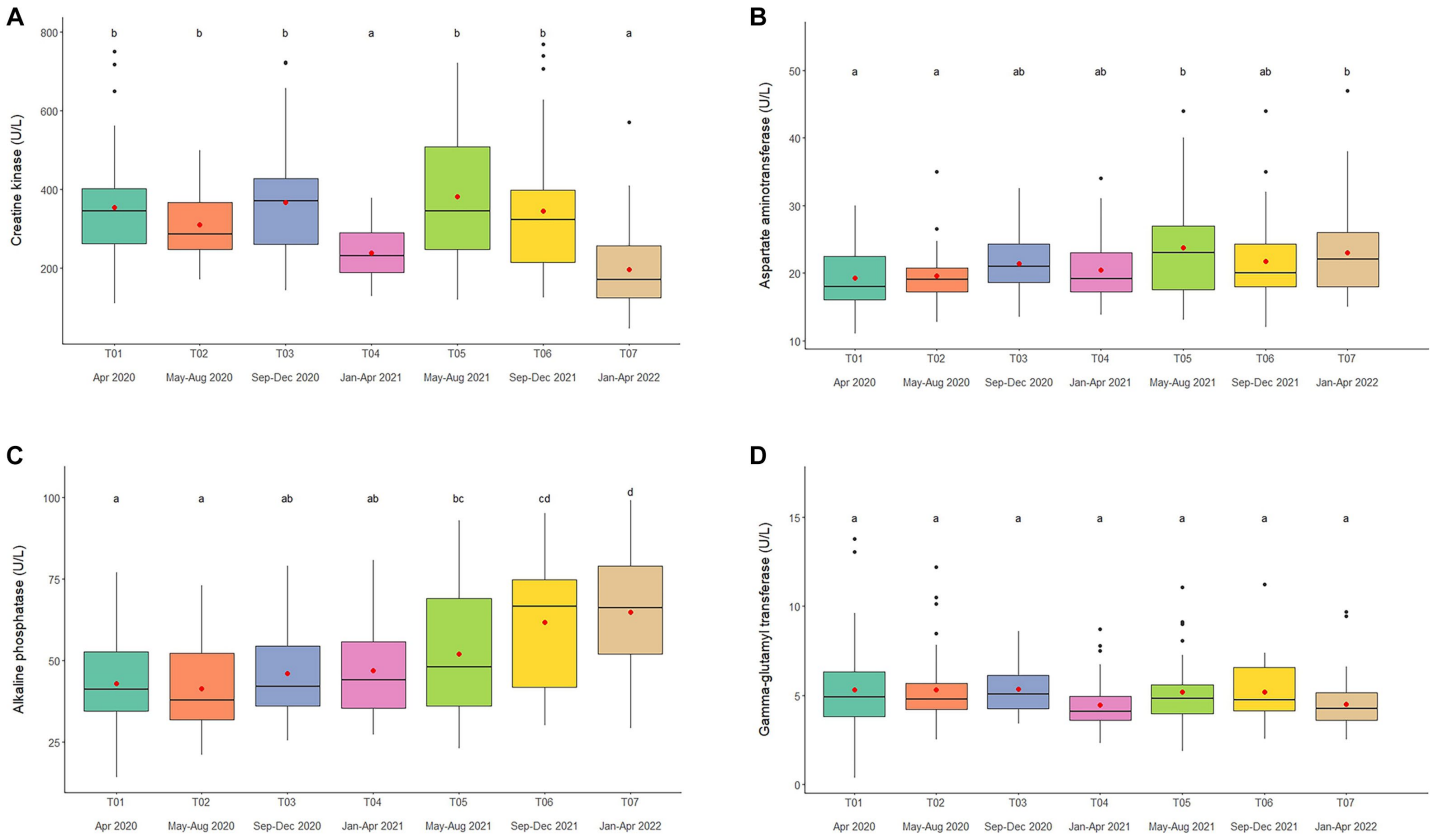
Boxplots of muscle and liver enzyme concentrations, **(A)** creatine kinase, **(B)** aspartate aminotransferase, **(C)** alkaline phosphatase, and **(D)** gamma-glutamyl transferase in captive Asian elephants (*n* = 58) over a 2-year period during the COVID-19 pandemic and international travel ban. Each plot shows the median (horizontal line), mean (red dot), 25 and 75% quartile ranges, and minimum and maximum ranges (whiskers). Different letters indicate significant differences between time periods (*p* < 0.001).

Overall average CK concentration was 320.4 ± 5.3 U/L (mean range, 196.7–387.3 U/L). Variables significantly affecting CK concentrations in the multivariate model were Time, Camp, and Chain hour (Tables 1, 2; Supplementary Table 3), with longer chaining hours having a significant positive effect (Tables 1, 2; Supplementary Table 3). Changing trends of CK were varied across time periods (Figure 2A; Table 2; Supplementary Tables 2, 3) and between camps (Table 2; Supplementary Tables 2, 3), with a declining trend over the 2-year period in four (Camp A, B, C, and E) of six camps (Supplementary Table 2), and lowest overall concentrations in T07 (Figure 2A; Table 2; Supplementary Tables 2, 3). There was a seasonal effect on CK, with higher concentrations in the rainy and winter seasons as compared to the summer months (Supplementary Table 16).

Overall average AST concentration was 21.4 ± 0.2 U/L (mean range, 19.1–24.4 U/L). Variables significantly affecting AST in the multivariate analysis were Time, Camp and Chain length (Tables 1, 2; Supplementary Table 4), with longer chaining lengths having a significant positive effect (Tables 1, 2; Supplementary Table 4). Average AST were varied by time (Figure 2B; Table 2; Supplementary Tables 2, 4) and between camp (Table 2; Supplementary Tables 2, 4). Concentrations increased over time in three camps (Camp A, E, and F) of six camps (Supplementary Table 2), reaching the highest concentration of significance at T05 (Figure 2B; Table 2; Supplementary Table 4). There was no relationship of AST to seasonal effects (Supplementary Table 16).

Overall average ALP concentration was 53.0 ± 0.8 U/L (mean range, 44.0–72.1 U/L). From the multivariate GEE analysis, factors that had a significant effect on ALP concentrations included Age, Time and Camp (Tables 1, 2; Supplementary Table 5), with negative relationship with age (Table 1; Supplementary Table 5). Average ALP concentrations were increased among five camps (Camp A–E) except Camp F (Supplementary Table 2) and increased over the 2-year period (Figure 2C; Table 2; Supplementary Tables 2, 5), being significant highest in T07 (Figure 2C; Table 2; Supplementary Table 5), but with no seasonal effect (Supplementary Table 16).

Overall average GGT concentration was 5.1 ± 0.1 U/L (mean range, 4.1–5.5 U/L). Factors affecting GGT concentrations in the multivariate model were Age and Camp (Tables 1, 2; Supplementary Table 6), with a positive relationship to Age (Table 1; Supplementary Table 6). There were no differences on time period, camp management (Figure 2D; Tables 1, 2; Supplementary Tables 2, 6) or seasonal (Supplementary Table 16) effects on GGT concentrations.

### Lipid profiles

**Figure 3:**
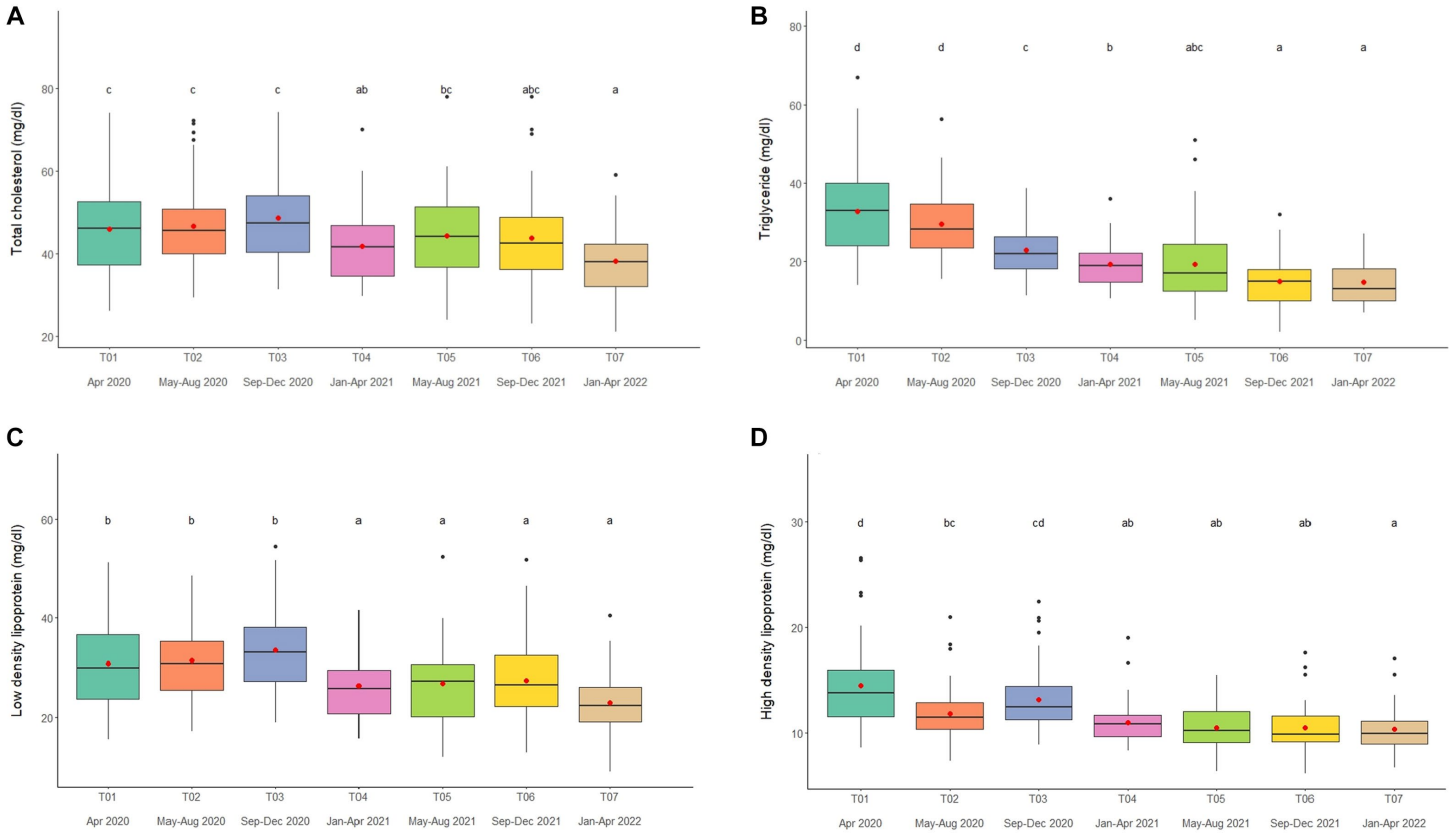
Boxplots of lipid profiles **(A)** total cholesterol, **(B)** triglycerides, **(C)** low density lipoproteins, and **(D)** high density lipoproteins in captive Asian elephants (*n* = 58) over a 2-year period during the COVID-19 pandemic and international travel ban. Each plot shows the median (horizontal line), mean (red dot), 25 and 75% quartile ranges, and minimum and maximum ranges (whiskers). Different letters indicate significant differences between time periods (*p* < 0.001).

Overall average TC concentration was 45.5 ± 0.5 mg/dL (mean range, 38.2–50.4 mg/dL). Variables significantly affecting TC in the multivariate analysis were Sex, Time, Camp and Roughage (Tables 1, 2; Supplementary Table 7) and higher in males than females (Tables 1, 2; Supplementary Table 7). There was a positive association with Roughage (Tables 1, 2; Supplementary Table 7). Average TC concentrations declined trend over the 2-year period in four camps (Camp A, B, C, and F) of six camps (Supplementary Table 2) and were lowest at T07 (Figure 3A; Table 2; Supplementary Table 2). There was no effect of season (Supplementary Table 16).

Overall average TG concentration was 22.9 ± 0.4 mg/dL (mean range, 14.7–32.8 mg/dL). From the multivariate analysis, Age, Time, Camp, Walking distance, Roughage and Supplement all affected TG concentrations (Tables 1, 2; Supplementary Table 8). Negative relationships were observed between TG and Age, Walking distance, and Supplement, with a positive relationship for Roughage (Tables 1, 2; Supplementary Table 8). A declining trend over time noted in four camps (Camp A, D, E, and F) of six camps (Supplementary Table 2), reaching the lowest concentration at T07 (Figure 3B; Table 2; Supplementary Tables 2, 8). TG concentrations were highest in the rainy season and lowest in summer (Supplementary Table 16).

Overall LDL concentration was 29.3 ± 0.3 mg/dL (mean range, 23.0–33.5 mg/dL). In the final multivariate GEE model, Sex, Time and Camp had significant effects on LDL (Tables 1, 2; Supplementary Table 9). Concentrations were higher in males than females (Tables 1, 2; Supplementary Table 9). A declining trend over time observed in four camps (Camp A, B, C, and F) of six camps (Supplementary Table 2). There was stable across seasons (Supplementary Table 16).

Overall mean HDL was 12.1 ± 0.1 mg/dL (mean range, 10.3–15.6 mg/dL). Variables significantly affecting HDL in the final GEE model were Time, Chain length and Chain hour (Tables 1, 2; Supplementary Table 10). Changing trends of HDL were varied across time periods (Figure 3D; Table 2; Supplementary Tables 2, 10), with a declining trend over the 2-year period in five camps (Camp B–F) of six camps (Supplementary Table 2). While, there were no differences between camps (Tables 1, 2; Supplementary Tables 2, 10). Longer chains lengths were associated with higher HDL concentrations, while chain hours had a negative effect (Table 1; Supplementary Table 10). HDL concentrations were lowest in the rainy and highest in the summer seasons (Supplementary Table 16).

### Plasma glucose, serum insulin, G:I, and serum fructosamine

**Figure 4:**
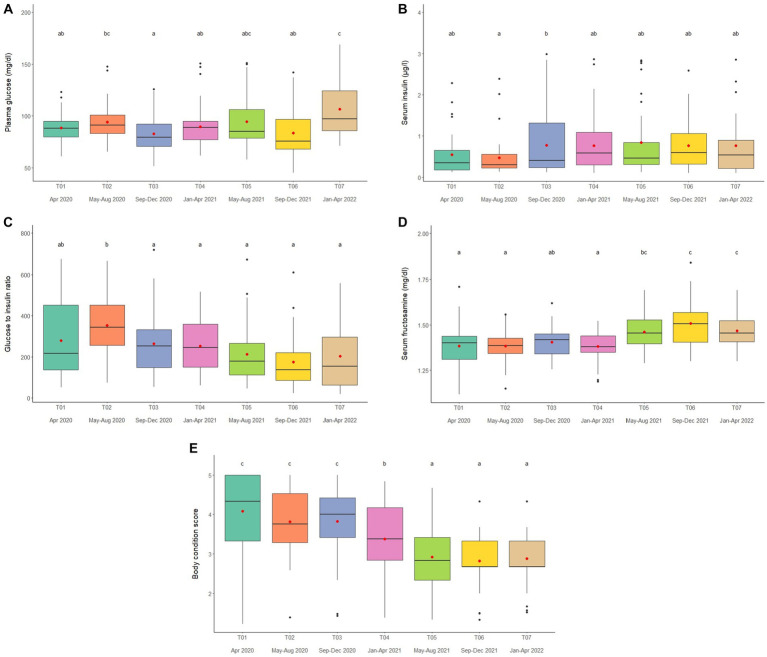
Boxplots of **(A)** plasma glucose, **(B)** serum insulin **(C)** glucose to insulin ratio **(D)** serum fructosamine and **(E)** body condition scores in captive Asian elephants (*n* = 58) over a 2-year period during the COVID-19 pandemic and international travel ban. Each plot shows the median (horizontal line), mean (red dot), 25 and 75% quartile ranges, and minimum and maximum ranges (whiskers). Different letters indicate significant differences between time periods (*p* < 0.001).

Overall average glucose concentration was 90.9 ± 0.8 mg/dL (mean range, 81.5–106.4 mg/dL). From the multivariate analysis, Time, Camp and Walking distance affected glucose concentrations (Tables 1, 2; Supplementary Table 11). Walking distance was positively related to plasma glucose concentrations. Changing trends of plasma glucose were varied across time periods (Figure 4A; Table 2; Supplementary Tables 2, 11) and between camps (Table 2; Supplementary Tables 2, 11), with an increasing trend over the 2-year period in three (Camp A, E, and F) of six camps, while gradually declined in only Camp B (Supplementary Table 2). Glucose was lowest in winter compared to summer and rainy seasons (Supplementary Table 16).

Serum insulin overall concentration was 0.83 ± 0.11 μg/L (mean range, 0.51–1.48 μg/L). In the final GEE model, Time, Camp and Walking distance were significant (Tables 1, 2; Supplementary Table 12). Walking distance exhibited a positive relationship to insulin (Table 1; Supplementary Table 12). Serum insulin was increased among three camps (A, E, and F) and decreased on Camp B (Supplementary Table 2), with the lowest concentration at T03 and the highest at the end of the study (T07) (Figure 4B; Table 2; Supplementary Tables 2, 12). There was no seasonal effect on insulin secretion (Supplementary Table 16).

Overall average G:I ratio was 274.2 ± 6.4 (mean range, 193.8–376.1). Factors affecting G:I ratio in the final GEE model were Time and Camp, but no camp management variables were associated with the G:I ratio (Tables 1, 2; Supplementary Table 13). G:I ratio were decreased over time; i.e., the highest G:I ratio was at T02 and lowest was at T06 (Figure 4C; Table 2; Supplementary Table 13), declining in three camps (Camp A, E, and F) of six camps (Supplementary Table 2). That coupled with the seasonal difference in plasma glucose resulted in a seasonal effect on the G:I ratio, with values of the rainy had higher than the winter season (Supplementary Table 16).

Overall average fructosamine concentration was 1.40 ± 0.01 mM (mean range, 1.41–1.54 mM). Results of the multivariate GEE analysis indicated Sex, Time and Walking distance were significant in the final model (Tables 1, 2; Supplementary Table 14). Concentrations of serum fructosamine were lower in females than males and negatively related to Walking distance (Table 1; Supplementary Table 14). Serum fructosamine were varied across camps (Figure 4D; Table 2; Supplementary Tables 2, 14) and increased over time in four camps (Camp A, B, C, and F) of six camps (Supplementary Table 2). Unlike glucose, serum fructosamine was not affected by season (Supplementary Table 16).

### Body condition scores

The overall median score was 3 (range, BCS = 1–5), with a mean of 3.6 ± 0.01 (range, BCS = 1–5). BCS decreased as the study progressed, with BCS = 5 observed in 44.8% of elephants at Time 1 and none at Time 7. Decreases were observed in all (Camp A, B, D, E, and F) but one (Camp C) camp (Figure 4E; Table 2; Supplementary Table 2), with elephants in Camp F becoming very thin by T07 (BCS = 2). In the final GEE multivariate model was related to Sex, Time, Camp and Supplement (Tables 1, 2; Supplementary Table 15). Overall, BCSs were lower in males than females (Table 2; Supplementary Table 15), and positively correlated to Supplements (Table 1; Supplementary Table 15). BCS remained stable throughout the year (Supplementary Table 16).

### Camp differences in physiological parameters

The results showed that all parameters except HDL and serum fructosamine had camp differences during the study of COVID-19 pandemic. For muscle and liver function parameters, Camp B had the highest, while Camps E had the lowest CK concentrations in our study. Camp F had the highest, while Camp B had the lowest AST concentrations that opposite with ALP, highest at Camp B and lowest at Camp F. Moreover, Camp D had the highest, while Camp C had the lowest GGT concentrations. For lipid profiles in metabolic function parameters, Camp A had the highest, while Camp D had lowest TC and LDL concentrations. Camp F had the highest, while Camp C had lowest TG concentrations. For metabolic markers, Camp A had highest, while Camp C lowest plasma glucose and serum insulin concentrations. In contrast, Camp C had highest, while Camp A had lowest G:I ratio. During the study, Camp B had highest, while Camp F had lowest BCS across times.

## Discussion

The international travel ban instituted in 2020 because of the SARS-CoV-2 (COVID-19) pandemic had significant effects on elephant tourist camp management in Thailand, in the form of reduced exercise opportunities and food provided, increased chaining time, and fewer mahouts (keepers) to care for the elephants (12) compared to previous years (2–5). This study used an epidemiological approach to examine how these changes affected physiological function based on measures of muscle, liver, lipid, and metabolic markers, and body condition over a 2-year period during the lockdown. Using T01 as a baseline 1 month into the travel ban, a number of changes were noted. The muscle enzyme, CK, was lowest at the end of the study after a prolonged period of physical inactivity. Increases in liver enzymes AST and ALP also were observed possibly due to changes in activity or nutrition. By contrast, reduced feeding of supplements (e.g., bananas, sugar cane), a main tourist attraction, was associated with fewer elephants scoring in overweight or obese BCS categories, despite the lack of exercise. As BCS declined, there were decreases observed in lipid markers (TC, TG, LDL, and HDL), implying some health benefits to feeding fewer high calorie treats. However, plasma glucose increased at the end of the study possibly due to the return of some local tourists. Overall, major changes in elephant management in the absence of tourists had significant effects on some health biomarkers and body condition, although patterns were not always consistent or easy to interpret.

### Muscle and liver enzymes

The reference range for serum CK in Asian elephants is 50–250 U/L (22), which is overall lower than that noted in this study (196.7–387.3 U/L). This enzyme is found in muscles and regarded as an indicator of cell activity, with increases associated with exercise (23–26), muscle damage (27, 28), and inflammation or trauma (29, 30). In this study, CK concentrations were highest at the beginning of the study and decreased over time in most elephants. Before the international travel ban, most elephants had been involved in riding or shows (12), which then abruptly ceased. Thus, higher concentrations at the beginning might reflect work activities, which then declined. This result agrees with human studies showing physical inactivity can reduce serum CK concentrations (20, 21, 29, 30). Of note was one camp with no change in CK concentrations (Camp F) that did not alter walking distance or chain hours, while two camps (Camp B and Camp D) that walked elephants the most before COVID-19 had higher initial values, which then declined. CK concentrations also were higher with longer chaining hours, suggesting muscle changes in association with prolonged restraint, standing or recumbency (31–33). Although exercise does not always increase CK, and there is considerable inter-individual variability (26, 34), reduced physical activity and standing for prolonged periods may have affected muscle enzyme activity in these elephants. More studies are needed to explore possible relationships between exercise, or lack thereof, and CK in elephants, especially as tourist activities return to normal.

In examining AST, concentrations were within the reported reference range for elephants (15–35 IU/L; (22)), but there was considerable variability in patterns among camps and time periods. AST is widely distributed, particularly in skeletal and cardiac muscles, the liver and erythrocytes (35), with elevations caused mainly by hepatocellular injuries (36). Our hypothesis was that AST concentrations would increase over time as elephants became more sedentary, similar to humans (37). That was observed at three camps (Camp A, C, and F); however, concentrations were unchanged in the other three (Camp B, D, and E). Effects of physical activity on AST are not straightforward, with some studies showing increases (38, 39) while others report decreases (37, 40, 41) in relation to exercise activity. In humans, elevated AST (and ALP) can occur in cases of starvation or malnutrition (42), due to decreased liver blood flow and hepatocyte degradation (43–46), but mechanisms are unclear (47). Many elephants lost body condition due to reduced feed intake, but scores for the vast majority did not indicate malnutrition. Rather, scores decreased from overweight/obese (BCS = 4/5) to normal (BCS = 3) categories, except for Camp F where there was, in fact, a dramatic decline in body condition (BCS = 1–2). In that camp, there also was a significant increase in AST over time, suggesting the two might be linked. Camp C was the only one where BCS did not change statistically, and neither did AST. In a study in Nepal, higher AST was found in one elephant that had a low BCS (48). Thus, there may be a relationship between diet changes and liver function in elephants, similar to human studies that have found an inverse association between weight loss and elevated liver enzymes (49–51).

For ALP, the normal range is 60–450 U/L (22), higher overall than that observed in this study (44.0–72.1 U/L). There was a clear and steady increase in ALP over the course of the study in all but one camp (Camp F). It is possible that physical inactivity may have contributed to elevated levels of ALP by affecting liver function (52), although there were no relationships between ALP and walking distance or chain hours across camps, or any other management factors included in the study. Nor were measures of GGT affected by any factors in the model except age.

### BCS and lipid profiles

In previous studies of this population, many elephants scored in overweight categories (BCS = 4, 27%; BCS = 5, 12%), whereas those participating in saddle or bareback riding were more likely to score an ideal BCS = 3 (5). In the U.S., elephants that walked more than 14 h/week had a decreased risk of BCS = 4 or 5 (53, 54), while higher feeding diversity (i.e., presenting food in multiple ways) also was related to lower BCSs (46, 48). An interesting finding in the U.S. study was that higher BCSs were related to training, presumed to be from the use of high-calorie treats as positive reinforcers (53). In this study, BCS was positively associated with amount of supplements fed, and elephants in T01 were even fatter than in previous studies (BCS = 1, 0%; BCS = 2, 1.7%; BCS = 3, 24.1%; BCS = 4, 29.3%; BCS = 5, 44.8%). During the travel ban, camp budgets were severely affected by the lack of tourist income and supplements were reduced by about half or more after the first year of the lockdown. This resulted in significant reductions in BCS, especially in the final time period where a number of elephants scored less than 3 (BCS = 1, 2.5%; BCS = 2, 17.5%; BCS = 3, 57.5%; BCS = 4, 22.5%; BCS = 5, 0%). Elephants at one camp (Camp F) became very thin (BCS = 2; range, 1–3); these elephants received the least amount of supplements. So, even though elephants were getting less exercise overall, they still lost significant body condition.

Lipid parameters (TC, TG, LDL and HDL) decreased over time in concert with reductions in BCS. Across camps, TC concentrations at the beginning were within the mean range previously reported (3–5) (24.4–48.5 mg/dL), but decreased by about 20% between T01 (range 36.0–64.1 mg/dL) and T07 (31.1–44.0 mg/dL). TG concentrations also declined steadily from ~33 to 15 mg/dL over the course of 2 years, reaching concentrations at the low end of previous reports (18.1–43.0 mg/dL) (3–5). The same was true for LDL (T01-T07, 32–23 mg/dL) and HDL (T01-T07, 14.5–10 mg/dL), declining over time and ending at the mid to lower end of previous ranges (LDL, 14.3–41.1 mg/dL; HDL, 9.2–19.0 mg/dL) (3–5). Before COVID-19, positive correlations were noted between BCS and several lipid markers (TC, TG, LDL, and HDL) (3–5). In this study, TG also was positively correlated to BCS, but the other lipids exhibited weak negative correlations. The positive correlation noted between TG and BCS is similar to that observed in African elephants (53) and horses (55, 56), as well as our earlier studies in Asian elephants (3–5). In healthy people, HDL is commonly referred to as good cholesterol, mitigating the risk of cardiovascular disease (57), and is generally negatively correlated to LDL and cholesterol (58, 59). However, it may not function the same way in elephants because TC, LDL, and HDL all were positively correlated in this and a prior study (5). Sex differences were observed for TC and LDL, with higher concentrations in males, which was somewhat surprising given that BCSs were lower in males. An age difference was observed only for TG, which agrees with another report in captive Asian elephants (60).

There were differences across camps in lipid profiles and some were related to diet or management, including walking distance (TG, negative), chain length (HDL, positive), chain hour (HDL, negative), roughage (TC, positive; TG, positive), and supplements (TG, negative). In previous studies, only walking distance was correlated to TC (positive) (3–5), perhaps reflecting differences between study years and the presence or absence of tourists (12). Exercise can increase HDL concentrations and decrease LDL, TG and TC in healthy people (61). In horses, an increase in TG was observed in response to jumping events (62–64), although no change in lipid profiles was observed in race horses (65). The higher HDL related to longer chain lengths and shorter chain hours in this study could reflect activity differences, although in reality, none of the elephants were getting much exercise during the COVID-19 lockdown. Roughage provided consisted mainly of corn stalk, napier grass (*Pennisetum purpureum*) and bana grass (*Pennisetum purpureum* X, *P. americanum* hybrid) (2–5, 61), and amount provided was positively related to lipids (TC and TG). Crude fat composition of napier grass and corn stalk ranges from 4.11–5.5% dry weight (61) and can vary depending on stage of plant maturity (66). In a previous analysis, primary diet (i.e., roughage) was correlated positively with BCS (and insulin), but not lipids (3). While it is not known if roughage quality was diminished due to budget cuts, at least amount fed appeared to affect lipid profiles in this study.

### Plasma glucose, serum insulin, G:I, and serum fructosamine

Overall plasma glucose (68.9–112.5 mg/dL), serum insulin (0.12–2.10 μg/L), and serum fructosamine (0.54–0.67 mM), concentrations were within normal ranges (3–5, 14, 22, 67), although plasma glucose and serum fructosamine, the latter of which is an assessment of long-term glycemic control in humans, fluctuated and were higher at the end of the study as some travel restrictions lifted. In relation to management, there were positive associations between glucose, insulin and fructosamine, but not G:I, with walking distance. These findings all agree with earlier work in this population (3). During exercise, skeletal muscle increases glucose uptake from the blood to meet the demand for carbohydrates as a source of energy (68). Thus, although activity levels were decreased during the travel ban, relations to metabolic activity were still present. In elephants, higher amounts of supplementary foods (banana, sugarcane) have been associated with higher glucose, fructosamine, and insulin concentrations (5), and an increase in plasma glucose and serum fructosamine was observed toward the end of the study as local tourists began to return after lifting some travel restrictions.

### Seasonal effects on physiological parameters.

There are three seasons in Thailand – summer (16 February–15 May), rainy (16 May–15 October) and winter (16 October–15 February), which have mild to moderate changes in temperature, rainfall, and humidity. While seasonal patterns in lipid and metabolic markers have been documented for tourist camp elephants in Thailand (3–5), data on muscle and liver function are new information. There was a seasonality in CK concentrations, being highest in the rainy and winter months, which contrasted with a study of Myanmar logging camp elephants, where CK concentrations were highest in the summer and lowest in the rainy season (69). However, in that study elephants also were not working due to a logging ban, so authors speculated the CK variation might reflect a natural seasonal variation in physiological processes rather than workload. While exposure to high temperatures can lead to dehydration and muscle damage as animals try to cool themselves (70, 71), elephants may also be susceptible to cold. Ambient temperatures in Thailand are cooler in the rainy and winter seasons, and some camp owners claim elephants shiver when they are cold. In humans, cold exposure is associated with increased levels of CK (72), and in rats, serum CK increased upon prolonged exposure to cold temperatures (73, 74). Thus, more studies are needed to tease apart environmental (i.e., seasonal) versus workload effects on CK in elephants. Other parameters (AST, ALP, and GGT) showed no seasonal variations over the 2-year study, in agreement with Myanmar elephant data (71).

For lipid and metabolic markers, comparing data with and without tourists, points of agreement included lower TG in summer for female elephants (5) and HDL in rainy season months for both males and females (3, 4). By contrast, lower TC in the rainy season was noted previously (5), but was unchanged in this study. For metabolic markers, our previous studies revealed plasma glucose was lowest in the rainy and highest in summer and winter seasons for both male (4) and female (5) elephants. Another evaluation found higher glucose concentrations in the high (November–February, winter months) versus low (March–October, summer and rainy months) tourist seasons (3), and was related to feeding high-calorie supplements (3). By contrast, our study found the opposite - lower concentrations in the winter and higher concentrations in the rainy and summer seasons, which appears to reinforce an effect of feeding bananas and sugar cane on glucose metabolism. However, although serum insulin was highest in the winter together with glucose in prior studies (4, 5), no differences were found in this study, nor did season have a significant effect on G:I, either past or present.

No seasonal changes in body condition were noted in this study. Higher BCSs have been observed in the winter, overlapping with the high tourist season in one (5), but not all of our studies in Thailand (3, 4). In one study of eight tourist elephants at the National Elephant Institute in Lampang, 90 km south of Chiang Mai, BCSs were lower in the winter compared to summer and rainy seasons (75). That facility provided free forage opportunities and incorporated low-calorie foods such as corn and carrots into the routine feeding of supplements by visitors, potentially explaining seasonal BCS differences between studies. Free-ranging elephants in India exhibited a distinct pattern of lower BCS during the winter dry season, which increases in the shift from dry to wet seasons, suggesting a seasonal pattern driven by energy and resource availability (76). Similarly, peak body weight in logging elephants in Myanmar were observed during the monsoon season (July–October) (77). Thus, while body condition often is related to rainfall and resource access in free-ranging Asian elephants, patterns can become obscured by management practices for captive, working elephants.

## Conclusion

This is the first study to examine variation among camps in management responses to the COVID-19 pandemic, and how those changes impacted physiological function in elephants. Although there were some notable effects on some muscle and liver enzymes, lipids, and body condition, for other parameters, patterns were not always clear. One aim was to identify camps that may have adapted to meet elephant welfare needs in the absence of tourists. However, none can be considered successes based on increased times elephants spent on chains and limited walking opportunities. It should be noted that many of these problems existed before the pandemic (2, 7, 12), but they were exacerbated by the loss of tourists and associated income. Better plans are needed to prepare for possible future disruptions in tourism. These could include camps growing their own fodder or being located near forests where elephants can forage, the latter of which can only happen with government and community support. Elephant numbers should be limited and based on available space or adjoining land to permit free roaming or use of longer chains (20–30 m). Finally, as a social species, elephants must be allowed to socialize with compatible elephants for the majority of the day and not separated by chains. These adaptations are needed and could ensure better welfare for elephants, not just during a pandemic, but going forward once tourism returns in full.

## Data availability statement

The original contributions presented in the study are included in the article/Supplementary material, further inquiries can be directed to the corresponding author.

## Ethics statement

The studies involving humans were approved by the Human Research Ethics Committee (HS1/2564). The studies were conducted in accordance with the local legislation and institutional requirements. Written informed consent for participation in this study was provided by the participants’ legal guardians/next of kin. The animal studies were approved by This study was approved by the Institutional Animal Care and Use Committee, Faculty of Veterinary Medicine (FVM), Chiang Mai University (CMU), Chiang Mai, Thailand (FVM-ACUC; S4/2564). The studies were conducted in accordance with the local legislation and institutional requirements. Written informed consent was obtained from the owners for the participation of their animals in this study.

## Author contributions

JS: Conceptualization, Formal analysis, Investigation, Methodology, Validation, Writing – review & editing, Visualization, Writing – original draft. JB: Formal analysis, Methodology, Validation, Writing – review & editing, Conceptualization, Funding acquisition, Resources. PB: Conceptualization, Formal analysis, Investigation, Methodology, Writing – review & editing. CT: Conceptualization, Funding acquisition, Resources, Writing – review & editing. VP: Methodology, Software, Validation, Writing – review & editing. KP: Methodology, Validation, Writing – review & editing. NS: Methodology, Validation, Writing – review & editing. PT: Methodology, Validation, Writing – review & editing. JK: Conceptualization, Data curation, Formal analysis, Funding acquisition, Investigation, Methodology, Resource, Project administration, Supervision, Validation, Visualization, Writing – original draft, Writing – review & editing.
