# Supplementary material for: Physiological changes in captive elephants in Northern Thailand as a result of the COVID-19 tourism ban – muscle, liver, metabolic function, and body condition
Source: Front Vet Sci. 2023 Dec 21;10:1303537. doi: 10.3389/fvets.2023.1303537 (PMC10764436; doi:10.3389/fvets.2023.1303537)
Supplement: Supplementary file 1 [file Data_Sheet_1.pdf]

Supplementary Material

Physiological Changes in Captive Elephants in Northern Thailand as a Result of the COVID-19 Tourism Ban – Muscle, Liver, Metabolic Function, and Body Condition

Jarawee Supanta[1,2], Janine L. Brown[2,3,4†], Pakkanut Bansiddhi[1,2,3], Chatchote Thitaram[1,2,3] Veerasak Punyapornwithaya[1], Khanittha Punturee[5], Nopphamas Somboon[6], Patcharapa Towiboon[2] and Jaruwan Khonmee[1,2,3†*]

***Correspondence:** Jaruwan Khonmee: jaruwan.khonmee@cmu.ac.th

†**Equal contribution:** These authors contributed equally to the work and share senior authorship.

1 Supplementary Data

The raw measurements are provided in:

- the mahout questionnaire sheet used to record information during sample collection

2 Supplementary Tables

Supplementary Table 1. Summary (mean ± SEM, range) of tourist, elephant and mahout numbers, and elephant management at six Thailand tourist camps during the COVID-19 pandemic and international travel ban between April 2020 – April 2022. Yearly data are compared to pre-COVID-19 (T00) as described in Supanta et al. (12).

**Parameters**	**Time**	**Camp A**	**Camp B**	**Camp C**	**Camp D**	**Camp E**	**Camp F**
Participating elephant age (years)		32.2 ± 2.7 (19.0-50.0)	37.9 ± 2.9 (22.0-56.0)	35.4 ± 3.3 (20.0-56.0)	40.3 ± 2.6 (28.0-54.0)	45.2 ± 2.8 (40.0-56.0)	43.3 ± 6.7 (30.0-50.0)
Mahout age (years)		35.7 ± 2.2 (25.0-48.0)	35.7 ± 1.8 (23.0-47.0)	42.3 ± 3.2 (18.0-58.0)	30.9 ± 2.0 (23.0-40.0)	44.2 ± 3.4 (36.0-51.0)	25.7 ± 4.7 (20.0-35.0)
Tourist number/day	T00 (Before COVID-19)	200	600	300	600	50	15
	T01 (Apr 2020)	0	4	2	4	4	0
	T04 (Jan-Apr 2021)	0	4	2	4	4	0
	T07 (Jan-Apr 2022)	6	10	6	10	10	7
Mahout number/camp	T00 (Before COVID-19)	40	66	66	12	10	3
	T01 (Apr 2020)	40	54	66	12	10	3
	T04 (Jan-Apr 2021)	20	28	39	7	5	3
	T07 (Jan-Apr 2022)	18	20	25	4	4	2
Elephant number/camp[1]	T00 (Before COVID-19)	13:30	18:36	26:41	0:12	0:12	0:3
	T01 (Apr 2020)	13:30	18:36	26:41	0:12	0:12	0:3
	T04 (Jan-Apr 2021)	13:41	18:31	26:34	0:8	0:7	0:3
	T07 (Jan-Apr 2022)	13:35	18:28	12:28	0:5	0:7	0:3
Participating elephants[1]	T01 (Apr 2020)	5:9	4:10	5:7	0:10	0:5	0:3
	T04 (Jan-Apr 2021)	5:9	2:10	4:7	0:8	0:5	0:3
	T07 (Jan-Apr 2022)	2:8	0:7	1:4	0:6	0:5	0:3
Chain time (h/day)	T00 (Before COVID-19)	16.0	16.0	16.0	16.0	17.0	18.0
	T01 (Apr 2020)	22.5	22.0	23.5	16.0	17.0	20.0

	T04 (Jan-Apr 2021)	22.6 ± 0.1 (22.5-23.3)	48.0	23.5	48.0	14.6 ± 0.2 (14.0-15.0)	20.0
	T07 (Jan-Apr 2022)	22.7 ± 0.2 (22.5-24.0)	24.0	23.5	24.0	14.6 ± 0.2 (14.0-15.0)	20.0
Chain length (m)	T00 (Before COVID-19)	2.0	3.0	1.5	3.0	5.0	3.0
	T01 (Apr 2020)	3.9 ± 0.7 (1.5-10.0)	3.0	1.5	3.0	1.2 ± 0.1 (1.0-1.5)	3.0
	T04 (Jan-Apr 2021)	4.3 ± 0.8 (1.5-10.0)	3.0	1.5	3.0	1.2 ± 0.1 (1.0-1.5)	2.0
	T07 (Jan-Apr 2022)	5.1 ± 1.0 (1.5-10.0)	3.0	2.5	3.0	1.2 ± 0.1 (1.0-1.5)	2.0
Walking distance (km/day)	T00 (Before COVID-19)	6.0	10.0	20.0	6.0	2.0	3.0
	T01 (Apr 2020)	1.5	5.6 ± 0.0 (0.0-6.0)	4.0	1.0	1.0	3.0
	T04 (Jan-Apr 2021)	1.4 ± 0.1 (0.8-1.5)	2.0	1.3 ± 0.0 (1.2-1.3)	0.5	0.5	3.0
	T07 (Jan-Apr 2022)	1.1 ± 0.2 (0.0-1.5)	2.0	2.8 ± 0.8 (2.0-6.0)	1.0	2.0	3.0
Amount of roughage (kg/day)	T00 (Before COVID-19)	200.0	120.0	180.0	120.0	180.0	300.0
	T01 (Apr 2020)	192.3 ± 2.8 (180.0-200.0)	139.1 ± 4.6 (120.0-150.0)	180.0	120.0	180.0	200.0
	T04 (Jan-Apr 2021)	190.0 ± 3.8 (165.0-200.0)	134.1 ± 6.8 (100.0-150.0)	150.0	120.0	180.0	150.0
	T07 (Jan-Apr 2022)	154.6 ± 3.1 (150.0-180.0)	131.8 ± 7.6 (100.0-150.0)	120.0	120.0	180.0	150.0
Amount of supplement (kg/day)	T00 (Before COVID-19)	20.0	25.0	25.0	25.0	25.0	15.0
	T01 (Apr 2020)	14.2 ± 2.1 (5.0-20.0)	25.0	25.0	25.0	15.0	20.0
	T04 (Jan-Apr 2021)	4.7 ± 0.2 (3.0-5.0)	8.3 ± 1.2 (1.0-10.0)	1.0	10.0	15.0	5.0
	T07 (Jan-Apr 2022)	10.2 ± 1.8 (1.0-15.0)	11.9 ± 2.1 (1.0-15.0)	10.0	10.0	15.0	5.0

[1]Number of males:females

[a]Elephants were chained for 48 consecutive hours

Supplementary Table 2. Time and camp differences in muscle and liver enzymes, lipid profiles, metabolic function and body condition scores (BCS) (mean ± SEM, range) in captive Asian elephants (n = 58) in six Thailand tourist camps during the COVID-19 pandemic international travel ban each year between April 2020 – April 2022.

Parameters	Time	Camp A	Camp B	Camp C	Camp D	Camp E	Camp F
**Muscle and liver enzymes**							
Creatine kinase (U/L)	T01 (Apr 2020)	351.0 ± 40.5$^{ab, z}$ (110.0-751.0)	360.0 ± 24.0$^{b, y}$ (206.0-546.0)	405.0 ± 31.2$^{b, y}$ (260.0-650.0)	372.0 ± 47.5$^{ab, x}$ (198.0-718.0)	269.0 ± 39.9$^{ab, y}$ (173.0-433.0)	233.0 ± 31.6$^{a, x}$ (166.0-300.0)
	T04 (Jan-Apr 2021)	226.0 ± 13.4$^{ab, y}$ (127.8-311.3)	292.0 ± 17.6$^{c, xy}$ (201.8-378.0)	190.0 ± 12.5$^{a, x}$ (100.7-255.0)	257.0 ± 19.8$^{bc, x}$ (168.0-323.5)	232.0 ± 25.3$^{abc, y}$ (138.0-290.8)	179.0 ± 14.8$^{a, x}$ (151.5-213.0)
	T07 (Jan-Apr 2022)	101.0 ± 17.2$^{a, x}$ (45.0-241.0)	272.0 ± 27.7$^{b, x}$ (133.0-382.0)	225.0 ± 29.6$^{b, x}$ (113.0-311.0)	292.0 ± 62.9$^{ab, x}$ (127.0-570.0)	101.0 ± 26.3$^{a, x}$ (53.0-212.0)	178.0 ± 28.5$^{ab, x}$ (130.0-246.0)
Aspartate aminotransferase (U/L)	T01 (Apr 2020)	20.2 ± 1.7$^{ab, x}$ (11.0-30.0)	20.6 ± 1.1$^{b, x}$ (15.0-29.0)	18.9 ± 1.4$^{ab, x}$ (13.0-30.0)	19.0 ± 1.2$^{ab, x}$ (15.0-28.0)	14.6 ± 1.2$^{a, x}$ (11.0-18.0)	17.7 ± 1.0$^{ab, x}$ (16.0-20.0)
	T04 (Jan-Apr 2021)	22.3 ± 1.3$^{b, x}$ (14.8-34.0)	17.5 ± 0.9$^{a, x}$ (12.0-23.7)	19.1 ± 0.8$^{ab, x}$ (14.3-23.0)	19.8 ± 0.8$^{ab, x}$ (17.3-24.0)	21.8 ± 2.9$^{ab, y}$ (13.8-31.0)	24.5 ± 1.8$^{b, y}$ (20.0-27.0)
	T07 (Jan-Apr 2022)	25.9 ± 1.7$^{b, y}$ (19.0-38.0)	17.4 ± 0.7$^{a, x}$ (15.0-21.0)	21.4 ± 1.9$^{ab, x}$ (16.0-28.0)	26.8 ± 4.5$^{b, x}$ (17.0-47.0)	20.4 ± 1.2$^{ab, xy}$ (16.0-23.0)	26.0 ± 2.2$^{b, y}$ (22.0-31.0)
Alkaline phosphatase (U/L)	T01 (Apr 2020)	40.1 ± 5.0$^{ab, x}$ (21.0-90.0)	48.2 ± 3.2$^{b, x}$ (31.0-99.0)	40.2 ± 3.9$^{ab, x}$ (14.0-65.0)	46.2 ± 3.7$^{ab, x}$ (32.0-71.0)	41.8 ± 3.2$^{ab, x}$ (28.0-48.0)	33.3 ± 3.4$^{a, x}$ (25.0-38.0)
	T04 (Jan-Apr 2021)	44.5 ± 4.9$^{ab, xy}$ (28.0-106.3)	50.8 ± 5.0$^{b, x}$ (31.0-80.8)	46.7 ± 3.6$^{b, xy}$ (27.0-66.8)	50.4 ± 4.8$^{b, x}$ (33.5-80.0)	45.4 ± 3.5$^{b, x}$ (36.8-57.3)	31.0 ± 1.7$^{a, x}$ (28.5-35.0)
	T07 (Jan-Apr 2022)	61.1 ± 5.8$^{a, y}$ (41.0-146.0)	71.2 ± 7.3$^{a, y}$ (52.0-128.0)	73.2 ± 9.01$^{a, y}$ (35.0-92.0)	57.8 ± 5.3$^{a, y}$ (37.0-108.0)	73.8 ± 7.3$^{a, y}$ (52.0-97.0)	47.0 ± 7.4$^{a, x}$ (29.0-58.0)
Gamma-glutamyl transferase (U/L)	T01 (Apr 2020)	5.4 ± 0.7$^{a, y}$ (3.6-13.8)	4.8 ± 0.6$^{a, x}$ (1.1-12.0)	6.0 ± 1.0$^{a, y}$ (2.6-13.8)	5.3 ± 1.0$^{a, x}$ (0.4-9.6)	4.1 ± 0.7$^{a, x}$ (1.7-5.2)	5.8 ± 1.0$^{a, x}$ (5.1-6.9)
	T04 (Jan-Apr 2021)	3.9 ± 0.4$^{a, x}$ (2.3-7.8)	4.6 ± 0.3$^{a, x}$ (3.1-6.7)	4.5 ± 0.5$^{a, xy}$ (3.0-7.7)	5.8 ± 0.6$^{a, x}$ (4.0-9.0)	5.1 ± 0.6$^{a, x}$ (3.1-6.7)	4.9 ± 0.2$^{a, x}$ (4.5-5.2)
	T07 (Jan-Apr 2022)	4.2 ± 0.2$^{ab, xy}$ (2.7-5.1)	4.5 ± 0.8$^{ab, x}$ (2.6-9.5)	3.2 ± 0.3$^{a, x}$ (2.6-4.2)	5.5 ± 0.8$^{b, x}$ (3.7-9.7)	4.8 ± 0.7$^{ab, x}$ (2.5-6.6)	4.9 ± 0.5$^{ab, x}$ (3.7-5.8)
**Lipid profiles**							
Total cholesterol (mg/dl)	T01 (Apr 2020)	59.0 ± 3.5$^{c, y}$ (37.0-124.0)	43.6 ± 2.5$^{ab, y}$ (32.0-68.0)	46.2 ± 2.1$^{b, y}$ (32.0-59.0)	35.3 ± 2.6$^{a, x}$ (26.0-51.0)	44.6 ± 2.5$^{ab, x}$ (36.0-51.0)	50.3 ± 0.3$^{bc, z}$ (50.0-51.0)
	T04 (Jan-Apr 2021)	48.6 ± 2.4$^{b, x}$ (35.3-70.0)	35.0 ± 1.4$^{a, x}$ (29.8-46.0)	41.4 ± 1.7$^{b, xy}$ (32.5-50.0)	37.9 ± 3.6$^{ab, x}$ (30.5-63.0)	45.1 ± 4.0$^{b, x}$ (34.5-56.3)	45.4 ± 0.7$^{b, y}$ (43.8-46.8)
	T07 (Jan-Apr 2022)	42.3 ± 3.0$^{b, x}$ (21.0-59.0)	31.1 ± 1.1$^{a, x}$ (27.0-36.0)	36.8 ± 1.8$^{ab, x}$ (31.0-43.0)	38.0 ± 3.0$^{ab, x}$ (29.0-51.0)	44.0 ± 3.0$^{b, x}$ (35.0-54.0)	34.0 ± 2.2$^{ab, x}$ (29.0-38.0)
Triglycerides (mg/dl)	T01 (Apr 2020)	39.2 ± 2.5$^{c, z}$ (22.0-55.0)	26.8 ± 2.5$^{ab, x}$ (15.0-45.0)	21.6 ± 1.9$^{a, x}$ (14.0-36.0)	31.6 ± 1.9$^{bc, y}$ (23.0-40.0)	55.0 ± 3.7$^{d, z}$ (42.0-67.0)	42.0 ± 4.0$^{cd, y}$ (33.0-50.0)
	T04 (Jan-Apr 2021)	22.8 ± 1.5$^{b, y}$ (13.5-36.0)	19.5 ± 1.5$^{ab, x}$ (10.5-27.8)	17.1 ± 1.1$^{a, x}$ (11.5-22.0)	16.1 ± 1.4$^{a, x}$ (10.8-24.0)	17.2 ± 0.9$^{a, y}$ (14.8-21.0)	19.2 ± 4.5$^{ab, x}$ (11.0-29.8)

	T07<br>(Jan-Apr 2022)	10.9 ± 1.0$^{a, x}$<br>(7.0-16.0)	16.6 ± 2.5$^{ab, x}$<br>(9.0-25.0)	18.2 ± 2.4$^{b, x}$<br>(12.0-25.0)	17.7 ± 2.2$^{b, x}$<br>(10.0-27.0)	13.0 ± 1.3$^{ab, x}$<br>(10.0-18.0)	14.3 ± 4.0$^{ab, x}$<br>(8.0-24.0)
Low density lipoprotein (mg/dl)	T01<br>(Apr 2020)	47.6 ± 4.2$^{c, y}$<br>(21.7-75.2)	29.1 ± 2.0$^{ab, y}$<br>(20.3-46.9)	29.9 ± 1.5$^{ab, y}$<br>(19.6-37.1)	24.4 ± 2.3$^{a, x}$<br>(15.4-38.2)	33.6 ± 2.4$^{b, x}$<br>(25.6-40.2)	32.4 ± 1.5$^{b, y}$<br>(29.2-35.7)
	T04<br>(Jan-Apr 2021)	31.0 ± 1.5$^{b, x}$<br>(20.6-41.4)	21.7 ± 1.1$^{a, xy}$<br>(15.6-28.1)	25.8 ± 1.3$^{ab, xy}$<br>(20.1-33.9)	23.9 ± 2.8$^{ab, x}$<br>(17.1-43.3)	28.7 ± 2.8$^{ab, x}$<br>(20.4-36.6)	29.0 ± 0.3$^{b, y}$<br>(28.3-29.5)
	T07<br>(Jan-Apr 2022)	26.1 ± 2.5$^{b, x}$<br>(8.9-40.5)	17.7 ± 0.8$^{a, x}$<br>(15.6-21.5)	22.4 ± 1.2$^{b, x}$<br>(18.4-26.2)	21.4 ± 1.8$^{ab, x}$<br>(15.0-26.6)	27.0 ± 2.4$^{b, x}$<br>(21.8-35.2)	21.9 ± 1.5$^{ab, x}$<br>(19.2-25.4)
High density lipoprotein (mg/dl)	T01<br>(Apr 2020)	14.4 ± 1.1$^{a, x}$<br>(9.5-33.2)	19.0 ± 1.1$^{b, y}$<br>(13.8-31.1)	11.6 ± 0.6$^{a, y}$<br>(8.6-15.9)	13.6 ± 0.9$^{a, y}$<br>(9.3-18.1)	13.1 ± 0.5$^{a, x}$<br>(11.8-14.7)	11.9 ± 0.8$^{a, xy}$<br>(10.8-13.9)
	T04<br>(Jan-Apr 2021)	11.9 ± 0.7$^{ab, x}$<br>(2.3-7.8)	9.8 ± 0.3$^{a, x}$<br>(3.1-6.7)	11.3 ± 0.3$^{b, y}$<br>(3.0-7.7)	10.4 ± 1.0$^{ab, x}$<br>(8.4-17.4)	11.2 ± 0.8$^{ab, xy}$<br>(3.1-6.7)	12.0 ± 1.2$^{ab, y}$<br>(4.4-5.2)
	T07<br>(Jan-Apr 2022)	11.3 ± 0.7$^{a, x}$<br>(8.8-15.5)	9.3 ± 0.5$^{a, x}$<br>(7.3-11.1)	9.3 ± 0.2$^{a, x}$<br>(8.5-9.9)	11.2 ± 1.2$^{a, xy}$<br>(8.8-17.0)	11.1 ± 0.7$^{a, x}$<br>(8.4-12.4)	8.3 ± 1.1$^{a, x}$<br>(6.7-10.9)
**Metabolic functions**							
Plasma glucose (mg/dl)	T01<br>(Apr 2020)	90.0 ± 2.3$^{bc, x}$<br>(78.0-113.0)	95.0 ± 3.8$^{c, y}$<br>(78.0-123.0)	79.2 ± 2.5$^{a, x}$<br>(61.0-92.0)	92.5 ± 3.7$^{c, x}$<br>(76.0-118.0)	89.0 ± 5.4$^{abc, x}$<br>(77.0-104.0)	75.3 ± 4.7$^{ab, x}$<br>(65.0-85.0)
	T04<br>(Jan-Apr 2021)	109.8 ± 5.5$^{c, y}$<br>(89.0-150.8)	87.6 ± 2.3$^{b, x}$<br>(77.0-101.0)	74.7 ± 1.6$^{a, x}$<br>(62.8-83.0)	93.2 ± 3.1$^{bc, x}$<br>(83.5-113.3)	75.6 ± 5.9$^{ab, x}$<br>(61.5-93.8)	70.7 ± 3.4$^{a, x}$<br>(63.0-77.3)
	T07<br>(Jan-Apr 2022)	124.8 ± 10.0$^{bc, y}$<br>(77.0-169.0)	94.3 ± 6.2$^{ab, xy}$<br>(79.0-132.0)	95.2 ± 13.5$^{abc, x}$<br>(71.0-154.0)	88.2 ± 2.3$^{a, x}$<br>(82.0-99.0)	117.6 ± 3.8$^{c, y}$<br>(107.0-131.0)	110.3 ± 10.6$^{abc, y}$<br>(87.0-132.0)
Serum insulin (µg/l)	T01<br>(Apr 2020)	0.38 ± 0.9$^{ab, x}$<br>(0.14-1.03)	0.91 ± 0.17$^{b, y}$<br>(0.18-1.82)	0.25 ± 0.06$^{a, x}$<br>(0.13-0.47)	0.62 ± 0.26$^{ab, xy}$<br>(0.12-2.29)	0.24 ± 0.05$^{a, x}$<br>(0.17-0.31)	0.33 ± 0.10$^{a, y}$<br>(0.15-0.52)
	T04<br>(Jan-Apr 2021)	1.13 ± 0.18$^{b, xy}$<br>(0.32-2.87)	0.94 ± 0.14$^{b, y}$<br>(0.53-3.34)	0.27 ± 0.03$^{a, x}$<br>(0.10-0.48)	1.00 ± 0.18$^{ab, y}$<br>(0.28-2.74)	0.52 ± 0.14$^{ab, x}$<br>(0.13-1.23)	0.19 ± 0.03$^{a, x}$<br>(0.12-0.23)
	T07<br>(Jan-Apr 2022)	1.30 ± 0.39$^{ab, y}$<br>(0.16-8.80)	0.25 ± 0.06$^{a, x}$<br>(0.10-6.75)	0.47 ± 0.22$^{ab, x}$<br>(0.16-0.78)	0.26 ± 0.03$^{a, x}$<br>(0.17-0.33)	1.03 ± 0.16$^{b, y}$<br>(0.54-1.54)	1.39 ± 0.47$^{ab, y}$<br>(0.73-2.06)
Glucose to insulin ratio	T01<br>(Apr 2020)	362.0 ± 64.3$^{ab, y}$<br>(88.0-614.4)	166.0 ± 37.5$^{a, x}$<br>(62.0-164.0)	435.0 ± 83.0$^{b, y}$<br>(154.4-674.6)	233.0 ± 53.1$^{ab, x}$<br>(51.6-706.7)	238.0 ± 71.3$^{ab, xy}$<br>(137.5-339.3)	287.0 ± 84.6$^{ab, y}$<br>(164.8-493.3)
	T04<br>(Jan-Apr 2021)	188.0 ± 26.5$^{a, x}$<br>(60.6-362.8)	172.0 ± 32.5$^{a, x}$<br>(62.0-403.1)	342.0 ± 28.7$^{b, x}$<br>(202.2-739.4)	284.0 ± 53.6$^{ab, x}$<br>(86.5-506.2)	305.0 ± 50.5$^{ab, z}$<br>(199.5-516.4)	374.0 ± 14.8$^{b, z}$<br>(353.4-782.0)
	T07<br>(Jan-Apr 2022)	127.0 ± 48.9$^{a, xy}$<br>(19.2-518.7)	217.0 ± 67.2$^{ab, x}$<br>(19.6-932.9)	557.0 ± 0.0$^{c, z}$<br>(103.8-756.9)	375.0 ± 45.1$^{b, y}$<br>(247.4-521.9)	127.0 ± 18.2$^{a, x}$<br>(85.0-200.1)	118.0 ± 45.2$^{a, x}$<br>(54.3-182.2)
Serum fructosamine (mM)	T01<br>(Apr 2020)	1.39 ± 0.03$^{a, x}$<br>(1.20-1.59)	1.37 ± 0.02$^{a, x}$<br>(1.26-1.54)	1.35 ± 0.05$^{a, x}$<br>(1.12-1.49)	1.41 ± 0.05$^{a, x}$<br>(1.14-1.60)	1.44 ± 0.07$^{a, x}$<br>(1.24-1.71)	1.34 ± 0.05$^{a, x}$<br>(1.22-1.41)
	T04<br>(Jan-Apr 2021)	1.42 ± 0.03$^{a, x}$<br>(1.23-1.52)	1.35 ± 0.01$^{a, x}$<br>(1.25-1.40)	1.36 ± 0.02$^{a, x}$<br>(1.20-1.46)	1.34 ± 0.03$^{ac, x}$<br>(1.19-1.51)	1.42 ± 0.03$^{a, x}$<br>(1.38-1.61)	1.41 ± 0.02$^{a, x}$<br>(1.38-1.45)
	T07<br>(Jan-Apr 2022)	1.51 ± 0.04$^{ab, y}$<br>(1.30-1.69)	1.43 ± 0.02$^{a, y}$<br>(1.37-1.50)	1.44 ± 0.02$^{a, x}$<br>(1.38-1.50)	1.43 ± 0.04$^{a, x}$<br>(1.31-1.61)	1.46 ± 0.04$^{ab, x}$<br>(1.30-1.52)	1.54 ± 0.01$^{b, y}$<br>(1.52-1.57)
Body condition score (1-5)	T01<br>(Apr 2020)	4.1 ± 0.2$^{a, z}$<br>(3.0-5.0)	4.4 ± 0.2$^{a, y}$<br>(3.0-5.0)	3.6 ± 0.2$^{a, x}$<br>(2.5-5.0)	4.4 ± 0.2$^{a, y}$<br>(4.0-5.0)	4.4 ± 0.2$^{a, y}$<br>(3.0-5.0)	2.5 ± 1.0$^{a, y}$<br>(3.0-5.0)
	T04<br>(Jan-Apr 2021)	3.2 ± 0.1$^{bc, y}$<br>(2.0-5.0)	4.1 ± 0.2$^{d, xy}$<br>(2.0-5.0)	3.1 ± 0.2$^{b, x}$<br>(2.0-5.0)	3.9 ± 0.2$^{cd, y}$<br>(2.0-4.0)	3.2 ± 0.4$^{abcd, y}$<br>(3.0-4.0)	1.8 ± 0.3$^{a, x}$<br>(1.0-3.0)
	T07<br>(Jan-Apr 2022)	2.8 ± 0.2$^{b, x}$<br>(2.0-4.0)	3.4 ± 0.2$^{b, x}$<br>(3.0-4.0)	3.0 ± 0.4$^{b, x}$<br>(2.0-4.0)	2.9 ± 0.2$^{b, x}$<br>(3.0-5.0)	2.8 ± 0.2$^{b, x}$<br>(2.0-3.0)	1.6 ± 0.0$^{a, x}$<br>(1.0-3.0)

^{a,b,c,d}Values in the same row are significantly different across camps (P <0.001).

^{x,y,z}Values in the same column are significantly different across time periods (P <0.001).

Supplementary Table 3. Univariate and multivariate GEE analyses of demographic and camp management variables associated with serum creatine kinase concentrations.

Variables		N	Univariate analysis			Multivariate analysis		
			**Estimate**	**SE**	**P value**	**Estimate**	**SE**	**P value**
**Sex**								
	Male	14	Reference					
	Female	44	5.780	22.100	0.790			
**Age**			-0.360	0.882	0.680			
**Time**								
	T01	58	Reference					
	T02	54	-44.200	20.600	0.032	-44.191	19.682	0.025
	T03	51	13.000	25.500	0.611	28.946	33.948	0.394
	T04	46	-116.700	19.200	<0.001	-100.809	34.192	0.003
	T05	40	27.300	32.000	0.395	11.844	38.994	0.761
	T06	38	7.600	36.700	0.836	13.882	42.613	0.745
	T07	36	-157.900	25.200	<0.001	-134.898	30.614	<0.001
**Camp**								
	A	14	57.500	22.500	0.011	9.415	24.141	0.103
	B	14	149.500	23.700	<0.001	116.735	26.061	<0.001
	C	12	72.200	23.700	0.002	58.208	23.896	0.015
	D	10	117.400	25..900	<0.001	132.002	33.814	<0.001
	E	5	-32.400	22.000	0.141	-11.282	28.042	0.687
	F	3	Reference					
**Walking distance (km/day)**			13.300	3.880	0.001	2.496	6.519	0.702
**Chain length (m)**			11.170	5.970	0.061	3.128	5.974	0.601
**Chain hour (h/day)**			6.400	1.130	<0.001	3.488	1.350	0.010
**Roughage (kg/day)**			-0.883	0.291	0.002	0.553	0.418	0.186
**Supplement (kg/day)**			2.502	0.954	0.009	0.261	1.786	0.884

SE =Standard error

Variables having a P value <0.15 at the univariate analysis were included in the multivariate analysis.

Supplementary Table 4. Univariate and multivariate GEE analyses of demographic and camp management variables associated with aspartate aminotransferase activity.

Variables		N	Univariate analysis			Multivariate analysis		
			Estimate	SE	P value	Estimate	SE	P value
**Sex**								
	Male	14	Reference					
	Female	44	-2.742	0.863	0.002	-1.852	0.971	0.057
**Age**			-0.054	0.029	0.059	0.010	0.027	0.714
**Time**								
	T01	58	Reference					
	T02	54	0.314	0.809	0.698	0.226	0.785	0.774
	T03	51	2.227	0.847	0.009	2.623	1.200	0.029
	T04	46	1.176	0.925	0.203	1.904	1.446	0.188
	T05	40	5.251	1.472	<0.001	5.189	1.516	0.001
	T06	38	4.494	1.292	0.042	3.244	1.438	0.024
	T07	36	3.804	1.308	0.004	4.467	1.294	0.001
**Camp**								
	A	14	-1.440	1.163	0.216	-2.962	1.050	0.005
	B	14	-4.201	1.074	<0.001	-4.928	1.331	<0.001
	C	12	-2.454	1.207	0.042	-2.049	1.314	0.119
	D	10	-2.753	1.250	0.028	-3.127	1.390	0.024
	E	5	-4.738	1.287	<0.001	-4.059	1.222	<0.001
	F	3	Reference					
**Walking distance (km/day)**			-0.188	0.144	0.190			
**Chain length (m)**			0.883	0.242	<0.001	0.837	0.352	0.017
**Chain hour (h/day)**			0.050	0.043	0.240	-0.008	0.060	0.890
**Roughage (kg/day)**			-0.005	0.011	0.660			
**Supplement (kg/day)**			-0.188	0.038	<0.001	0.040	0.078	0.604

SE =Standard error

Variables having a P value <0.15 at the univariate analysis were included in the multivariate analysis.

Supplementary Table 5. Univariate and multivariate GEE analyses of demographic and camp management variables associated with alkaline phosphatase activity.

Variables		N	Univariate analysis			Multivariate analysis		
			Estimate	SE	P value	Estimate	SE	P value
**Sex**								
	Male	14	Reference					
	Female	44	-3.280	2.610	0.250			
**Age**			-0.572	0.101	<0.001	-0.688	0.085	<0.001
**Time**								
	T01	58	Reference					
	T02	54	-1.440	2.620	0.583	-1.168	2.272	0.607
	T03	51	3.130	2.580	0.225	3.414	3.297	0.300
	T04	46	4.100	2.920	0.161	3.638	3.808	0.339
	T05	40	9.120	3.660	0.013	8.379	4.206	0.046
	T06	38	18.890	3.810	<0.001	18.965	4.020	<0.001
	T07	36	22.040	3.760	<0.001	23.660	3.944	<0.001
**Camp**								
	A	14	11.690	3.070	<0.001	4.547	2.676	0.089
	B	14	18.090	3.090	<0.001	18.388	3.293	<0.001
	C	12	12.860	3.000	<0.001	9.833	2.615	<0.001
	D	10	17.150	3.330	<0.001	20.714	4.160	<0.001
	E	5	16.900	3.830	<0.001	19.431	3.767	<0.001
	F	3	Reference					
**Walking distance (km/day)**			-0.980	0.509	0.054	0.147	0.765	0.848
**Chain length (m)**			0.877	0.796	0.270			
**Chain hour (h/day)**			0.441	0.153	0.004	0.249	0.154	0.107
**Roughage (kg/day)**			-0.132	0.040	<0.001	0.091	0.052	0.078
**Supplement (kg/day)**			-0.321	0.118	0.007	-0.119	0.193	0.538

SE =Standard error

Variables having a P value <0.15 at the univariate analysis were included in the multivariate analysis.

Supplementary Table 6. Univariate and multivariate GEE analyses of demographic and camp management variables associated with gamma-glutamyl transferase activity.

Variables		N	Univariate analysis			Multivariate analysis		
			Estimate	SE	P value	Estimate	SE	P value
**Sex**								
	Male	14	Reference					
	Female	44	-0.333	0.343	0.330			
**Age**			0.038	0.010	<0.001	0.040	0.012	<0.001
**Time**								
	T01	58	Reference					
	T02	54	0.033	0.443	0.942	0.037	0.431	0.931
	T03	51	0.051	0.406	0.900	0.097	0.417	0.816
	T04	46	-0.836	0.417	0.045	-0.801	0.416	0.054
	T05	40	-0.117	0.470	0.804	-0.101	0.474	0.831
	T06	38	-0.097	0.457	0.832	0.030	0.489	0.951
	T07	36	-0.799	0.453	0.078	-0.728	0.483	0.131
**Camp**								
	A	14	-0.337	0.287	0.240	0.069	0.352	0.845
	B	14	-0.251	0.298	0.401	-0.015	0.338	0.965
	C	12	-0.576	0.340	0.090	-0.284	0.381	0.456
	D	10	0.663	0.375	0.077	0.948	0.421	0.024
	E	5	-0.453	0.307	0.139	-0.537	0.338	0.113
	F	3	Reference					
**Walking distance (km/day)**			0.0160	0.070	0.820			
**Chain length (m)**			-0.011	0.061	0.850			
**Chain hour (h/day)**			0.005	0.012	0.660			
**Roughage (kg/day)**			-0.007	0.004	0.140	0.003	0.006	0.614
**Supplement (kg/day)**			0.019	0.017	0.240			

SE =Standard error

Variables having a P value <0.15 at the univariate analysis were included in the multivariate analysis.

Supplementary Table 7. Univariate and multivariate GEE analyses of demographic and camp management variables associated with total cholesterol concentrations.

Variables		N	Univariate analysis			Multivariate analysis		
			Estimate	SE	P value	Estimate	SE	P value
**Sex**								
	Male	14	Reference					
	Female	44	-8.000	1.570	<0.001	-4.169	1.464	0.004
**Age**			-0.061	0.063	0.330			
**Time**								
	T01	58	Reference					
	T02	54	0.706	2.083	0.734	-0.632	1.684	0.707
	T03	51	2.673	2.142	0.212	1.356	2.518	0.590
	T04	46	-4.126	2.028	0.042	-6.278	2.723	0.021
	T05	40	-1.694	2.292	0.460	-2.200	3.041	0.469
	T06	38	-2.208	2.456	0.369	-3.237	2.771	0.243
	T07	36	-7.750	2.052	<0.001	-8.233	2.379	<0.001
**Camp**								
	A	14	4.030	2.350	0.087	0.727	2.223	0.744
	B	14	-8.560	2.190	<0.001	-8.274	2.141	<0.001
	C	12	-1.440	2.210	0.515	-2.895	2.021	0.152
	D	10	-9.930	2.280	<0.001	-8.570	2.572	<0.001
	E	5	1.110	2.690	0.679	1.012	2.639	0.701
	F	3	Reference					
**Walking distance (km/day)**			-0.446	0.308	0.150	-0.279	0.398	0.484
**Chain length (m)**			1.040	0.420	0.013	0.680	0.402	0.091
**Chain hour (h/day)**			-0.202	0.063	0.001	-0.279	0.398	0.484
**Roughage (kg/day)**			0.190	0.021	<0.001	0.062	0.029	0.032
**Supplement (kg/day)**			-0.140	0.077	0.069	-0.044	0.135	0.746

SE =Standard error
Variables having a P value <0.15 at the univariate analysis were included in the multivariate analysis.

Supplementary Table 8. Univariate and multivariate GEE analyses of demographic and camp management variables associated with triglyceride concentrations.

Variables		N	Univariate analysis			Multivariate analysis		
			Estimate	SE	P value	Estimate	SE	P value
**Sex**								
	Male	14	Reference					
	Female	44	-1.340	1.450	0.350			
**Age**			-0.112	0.059	0.058	-0.169	0.043	<0.001
**Time**								
	T01	58	Reference					
	T02	54	-3.270	1.990	0.100	-3.268	1.632	0.045
	T03	51	-9.870	1.880	<0.001	-16.591	2.121	<0.001
	T04	46	-13.440	1.840	<0.001	-23.257	2.306	<0.001
	T05	40	-13.370	2.330	<0.001	-20.313	2.436	<0.001
	T06	38	-17.870	1.970	<0.001	-23.831	2.217	<0.001
	T07	36	-18.040	1.910	<0.001	-23.178	2.033	<0.001
**Camp**								
	A	14	-1.652	3.147	0.600	-6.439	2.180	0.003
	B	14	-0.464	3.135	0.880	3.811	2.246	0.090
	C	12	-4.550	3.010	0.130	-6.312	2.085	0.002
	D	10	-0.881	3.108	0.780	1.735	2.561	0.498
	E	5	-0.995	4.010	0.800	-0.132	2.680	0.961
	F	3	Reference					
**Walking distance (km/day)**			0.752	0.306	0.014	-0.908	0.466	0.050
**Chain length (m)**			0.448	0.369	0.230			
**Chain hour (h/day)**			-0.048	0.073	0.510			
**Roughage (kg/day)**			0.102	0.023	<0.001	0.095	0.0267	<0.001
**Supplement (kg/day)**			0.333	0.067	<0.001	-0.642	0.103	<0.001

SE =Standard error

Variables having a P value <0.15 at the univariate analysis were included in the multivariate analysis.

Supplementary Table 9. Univariate and multivariate GEE analyses of demographic and camp management variables associated with low density lipoprotein concentrations.

Variables		N	Univariate analysis			Multivariate analysis		
			**Estimate**	**SE**	**P value**	**Estimate**	**SE**	**P value**
**Sex**								
	Male	14	Reference					
	Female	44	-5.870	1.160	<0.001	-3.593	1.161	0.002
**Age**			-0.031	0.048	0.510			
**Time**								
	T01	58	Reference					
	T02	54	0.520	1.550	0.737	-2.455	1.497	0.101
	T03	51	2.620	1.670	0.117	-4.340	2.651	0.102
	T04	46	-4.440	1.450	0.002	-12.632	2.851	<0.001
	T05	40	-4.000	1.680	0.017	-10.417	2.875	<0.001
	T06	38	-3.430	1.810	0.058	-10.254	2.705	<0.001
	T07	36	-7.850	1.510	<0.001	-13.656	2.391	<0.001
**Camp**								
	A	14	2.946	1.752	0.093	1.659	1.653	0.316
	B	14	-6.677	1.649	<0.001	-5.444	1.600	<0.001
	C	12	-0.967	1.646	0.557	-2.031	1.498	0.175
	D	10	-7.036	1.733	<0.001	-7.115	1.909	<0.001
	E	5	1.615	2.103	0.442	2.362	2.050	0.249
	F	3	Reference					
**Walking distance (km/day)**			-0.300	0.252	0.230	-0.502	0.307	0.102
**Chain length (m)**			0.658	0.353	0.063	0.515	0.373	0.167
**Chain hour (h/day)**			-0.227	0.049	<0.001	-0.017	0.051	0.739
**Roughage (kg/day)**			0.139	0.016	<0.001	0.011	0.023	0.632
**Supplement (kg/day)**			-0.061	0.060	0.310			

SE =Standard error

Variables having a P value <0.15 at the univariate analysis were included in the multivariate analysis.

Supplementary Table 10. Univariate and multivariate GEE analyses of demographic and camp management variables associated with high density lipoprotein concentrations.

Variables		N	Univariate analysis			Multivariate analysis		
			Estimate	SE	P value	Estimate	SE	P value
**Sex**								
	Male	14	Reference					
	Female	44	-1.766	0.499	<0.001	-0.467	0.451	0.301
**Age**			-0.023	0.020	0.250			
**Time**								
	T01	58	Reference					
	T02	54	-2.649	0.659	<0.001	-2.815	0.622	<0.001
	T03	51	-1.352	0.695	0.052	-1.608	0.790	0.042
	T04	46	-3.516	0.638	<0.001	-3.556	0.769	<0.001
	T05	40	-4.031	0.666	<0.001	-3.347	0.782	<0.001
	T06	38	-3.976	0.679	<0.001	-3.416	0.719	<0.001
	T07	36	-4.143	0.666	<0.001	-4.047	0.694	<0.001
**Camp**								
	A	14	1.037	0.802	0.200			
	B	14	0.341	0.887	0.700			
	C	12	0.109	0.758	0.890			
	D	10	-0.632	0.818	0.440			
	E	5	0.288	0.788	0.710			
	F	3	Reference					
**Walking distance (km/day)**			0.376	0.144	0.009	0.213	0.126	0.090
**Chain length (m)**			0.475	0.110	<0.001	0.500	0.107	<0.001
**Chain hour (h/day)**			-0.086	0.015	<0.001	-0.071	0.015	<0.001
**Roughage (kg/day)**			0.022	0.006	<0.001	0.009	0.006	0.133
**Supplement (kg/day)**			0.051	0.028	0.066	-0.030	0.035	0.392

SE =Standard error

Variables having a P value <0.15 at the univariate analysis were included in the multivariate analysis.

Supplementary Table 11. Univariate and multivariate GEE analyses of demographic and camp management variables associated with plasma glucose concentrations.

**Variables**		**N**	**Univariate analysis**			**Multivariate analysis**		
			**Estimate**	**SE**	**P value**	**Estimate**	**SE**	**P value**
**Sex**								
	Male	14	Reference					
	Female	44	-0.489	3.081	0.870			
**Age**			-0.167	0.118	0.160			
**Time**								
	T01	58	Reference					
	T02	54	5.700	2.880	0.047	5.636	2.293	0.014
	T03	51	-5.620	3.070	0.067	-6.949	2.485	0.005
	T04	46	1.250	3.320	0.706	2.660	3.293	0.419
	T05	40	6.030	4.270	0.158	6.727	4.908	0.170
	T06	38	-4.770	4.070	0.241	-5.871	5.198	0.259
	T07	36	18.020	4.760	<0.001	16.336	4.346	<0.001
**Camp**								
	A	14	22.715	5.528	<0.001	27.871	5.517	<0.001
	B	14	5.560	5.069	0.273	3.091	5.131	0.547
	C	12	-0.429	5.229	0.935	1.419	4.763	0.766
	D	10	5.158	5.064	0.308	5.384	5.514	0.329
	E	5	13.362	6.286	0.034	17.898	6.121	0.003
	F	3	Reference					
**Walking distance (km/day)**			-1.013	0.599	0.091	2.162	0.923	0.019
**Chain length (m)**			1.964	0.807	0.015	0.269	0.921	0.770
**Chain hour (h/day)**			-0.354	0.098	<0.001	-0.170	0.150	0.259
**Roughage (kg/day)**			0.094	0.044	0.032	-0.125	0.069	0.068
**Supplement (kg/day)**			-0.155	0.132	0.240			

SE =Standard error

Variables having a P value <0.15 at the univariate analysis were included in the multivariate analysis.

Supplementary Table 12. Univariate and multivariate GEE analyses of demographic and camp management variables associated with serum insulin concentrations.

Variables		N	Univariate analysis			Multivariate analysis		
			Estimate	SE	P value	Estimate	SE	P value
**Sex**								
	Male	14	Reference					
	Female	44	0.056	0.113	0.620			
**Age**			-0.005	0.004	0.130	1.640	3.730	0.997
**Time**								
	T01	58	Reference					
	T02	54	-0.073	0.106	0.493	-6.140	1.060	0.563
	T03	51	0.227	0.136	0.096	3.560	1.540	0.021
	T04	46	0.226	0.131	0.084	4.500	2.010	0.025
	T05	40	0.299	0.172	0.083	4.890	2.140	0.022
	T06	38	0.218	0.140	0.121	4.420	1.970	0.025
	T07	36	0.223	0.167	0.182	3.400	1.830	0.064
**Camp**								
	A	14	0.757	0.138	<0.001	9.140	1.610	<0.001
	B	14	0.232	0.111	0.037	1.580	1.390	0.256
	C	12	-0.007	0.108	0.950	7.000	1.270	0.582
	D	10	0.214	0.126	0.090	4.240	1.520	0.005
	E	5	0.352	0.144	0.015	4.670	1.560	0.003
	F	3	Reference					
**Walking distance (km/day)**			-0.041	0.021	0.047	9.420	3.200	0.003
**Chain length (m)**			0.020	0.024	0.400			
**Chain hour (h/day)**			-0.004	0.003	0.140	-4.940	4.890	0.312
**Roughage (kg/day)**			0.001	0.001	0.485			
**Supplement (kg/day)**			-0.017	0.005	0.001	6.330	9.520	0.506

SE =Standard error

Variables having a P value <0.15 at the univariate analysis were included in the multivariate analysis.

Supplementary Table 13. Univariate and multivariate GEE analyses of demographic and camp management variables associated with glucose to insulin ratio.

Variables		N	Univariate analysis			Multivariate analysis		
			Estimate	SE	P value	Estimate	SE	P value
**Sex**								
	Male	14	Reference					
	Female	44	-48.500	33.300	0.150	-51.720	31.020	0.096
**Age**			0.909	1.050	0.390			
**Time**								
	T01	58	Reference					
	T02	54	71.700	36.100	0.047	65.430	34.920	0.061
	T03	51	-16.500	37.000	0.656	-64.700	49.410	0.190
	T04	46	-27.900	36.600	0.445	-78.620	54.910	0.152
	T05	40	-66.800	40.700	0.101	-95.090	49.580	0.055
	T06	38	-105.700	39.300	0.007	-135.560	48.770	0.005
	T07	36	-76.600	44.600	0.086	-93.240	48.040	0.052
**Camp**								
	A	14	-149.600	37.000	<0.001	-164.81	37.920	<0.001
	B	14	-91.500	38.600	0.018	-78.020	40.190	0.052
	C	12	12.000	39.700	0.763	-12.490	39.810	0.754
	D	10	-72.600	38.500	0.059	-67.000	44.800	0.135
	E	5	-98.500	42.500	0.020	-77.610	43.760	0.076
	F	3	Reference					
**Walking distance (km/day)**			15.390	6.470	0.017	-1.600	9.860	0.871
**Chain length (m)**			0.562	7.203	0.940			
**Chain hour (h/day)**			-1.246	0.901	0.170			
**Roughage (kg/day)**			0.294	0.385	0.444			
**Supplement (kg/day)**			2.590	1.390	0.062	-3.910	2.590	0.131

SE =Standard error

Variables having a P value <0.15 at the univariate analysis were included in the multivariate analysis.

Supplementary Table 14. Univariate and multivariate GEE analyses of demographic and camp management variables associated with serum fructosamine concentrations.

Variables		N	Univariate analysis			Multivariate analysis		
			Estimate	SE	P value	Estimate	SE	P value
**Sex**								
	Male	14	Reference					
	female	44	-0.021	0.014	0.130	-3.642	1.332	0.006
**Age**			0.000	0.001	0.700			
**Time**								
	T01	58	Reference					
	T02	54	9.110	1.832	0.996	3.233	1.762	0.854
	T03	51	2.042	1.822	0.264	3.582	2.392	0.134
	T04	46	-2.443	1.852	0.895	7.123	2.472	0.773
	T05	40	7.732	2.092	<0.001	8.572	2.782	0.002
	T06	38	1.241	2.532	<0.001	1.321	3.052	1.605
	T07	36	8.442	2.112	<0.001	9.472	2.492	<0.001
**Camp**								
	A	14	0.029	0.023	0.209	3.392	2.042	0.095
	B	14	0.030	0.024	0.212	3.602	2.222	0.104
	C	12	0.035	0.025	0.165	2.202	2.502	0.377
	D	10	0.036	0.025	0.153	3.582	2.312	0.121
	E	5	0.065	0.033	0.045	6.262	3.002	0.037
	F	3	Reference					
**Walking distance (km/day)**			0.011	0.003	<0.001	-7.533	3.253	0.020
**Chain length (m)**			0.004	0.003	0.290			
**Chain hour (h/day)**			0.002	0.001	0.001	4.524	8.504	0.595
**Roughage (kg/day)**			-0.000	0.000	0.094	7.265	2.384	0.761
**Supplement (kg/day)**			-0.002	0.001	0.010	1.423	1.293	0.270

SE =Standard error

Variables having a P value <0.15 at the univariate analysis were included in the multivariate analysis.

Supplementary Table 15. Univariate and multivariate GEE analyses of demographic and camp management variables associated with body condition scores.

Variables		N	Univariate analysis			Multivariate analysis		
			Estimate	SE	P value	Estimate	SE	P value
**Sex**								
	Male	14	Reference					
	Female	44	0.345	0.118	0.003	0.495	0.117	<0.001
**Age**			0.008	0.006	0.160			
**Time**								
	T01	58	Reference					
	T02	54	-0.265	0.163	0.100	-0.238	0.130	0.067
	T03	51	-0.251	0.170	0.140	-0.280	0.167	0.094
	T04	46	-0.700	0.179	<0.001	-0.724	0.183	<0.001
	T05	40	-1.157	0.181	<0.001	-1.176	0.184	<0.001
	T06	38	-1.255	0.163	<0.001	-1.237	0.168	<0.001
	T07	36	-1.197	0.177	<0.001	-1.204	0.162	<0.001
**Camp**								
	A	14	1.346	0.219	<0.001	1.509	0.238	<0.001
	B	14	1.988	0.224	<0.001	1.993	0.218	<0.001
	C	12	1.309	0.226	<0.001	1.270	0.237	<0.001
	D	10	2.021	0.238	<0.001	1.812	0.268	<0.001
	E	5	1.235	0.254	<0.001	1.289	0.265	<0.001
	F	3	Reference					
**Walking distance (km/day)**			0.113	0.029	<0.001	0.013	0.038	0.742
**Chain length (m)**			-0.024	0.023	0.300			
**Chain hour (h/day)**			-0.001	0.006	0.790			
**Roughage (kg/day)**			-0.004	0.002	0.089	-0.000	0.003	0.856
**Supplement (kg/day)**			0.045	0.006	<0.001	0.043	0.007	<0.001

SE =Standard error

Variables having a P value <0.15 at the univariate analysis were included in the multivariate analysis.

Supplementary Table 16. Seasonal effects on physiological parameters in Asian elephants. Mean ± SEM and range values for muscle and liver enzymes, lipid profiles, metabolic function and body condition scores (BCS) of captive Asian elephants (n = 58) in six Thailand tourist camps during the COVID-19 pandemic international travel ban each year between April 2020 – April 2022.

Parameters	Summer	Rainy	Winter
**Muscle and liver enzymes**			
Creatine kinase (U/L)	278.0 ± 12.2[a]<br>(130.6-531.2)	337.0 ± 15.4[b]<br>(161.2-578.5)	371.0 ± 21.6[b]<br>(136.4-797.4)
Aspartate aminotransferase (U/L)	20.7 ± 0.6[a]<br>(14.0-29.9)	21.5 ± 0.6[a]<br>(14.9-38.0)	22.1 ± 0.8[a]<br>(13.1-46.0)
Alkaline phosphatase (U/L)	51.8 ± 2.4[a]<br>(24.5-99.0)	48.9 ± 2.7[a]<br>(20.0-114.5)	56.3 ± 2.7[a]<br>(28.2-108.5)
Gamma-glutamyl transferase (U/L)	5.0 ± 0.3[a]<br>(0.4-12.0)	5.2 ± 0.2[a]<br>(2.8-10.1)	5.4 ± 0.2[a]<br>(3.1-9.4)
**Lipid profiles**			
Total cholesterol (mg/dl)	45.4 ± 1.6[a]<br>(26.0-80.7)	46.4 ± 1.2[a]<br>(29.9-69.3)	46.8 ± 1.4[a]<br>(29.6-76.1)
Triglycerides (mg/dl)	20.2 ± 1.4[a]<br>(13.5-38.0)	26.1 ± 1.9[b]<br>(12.9-46.5)	22.0 ± 1.7[ab]<br>(10.6-38.8)
Low density lipoprotein (mg/dl)	29.0 ± 1.1[a]<br>(15.8-51.1)	30.0 ± 1.0[a]<br>(16.8-45.6)	31.2 ± 1.1[a]<br>(16.4-50.5)
High density lipoprotein (mg/dl)	12.7 ± 0.4[b]<br>(8.8-26.6)	11.4 ± 0.3[a]<br>(8.0-18.0)	12.2 ± 0.4[ab]<br>(8.3-19.3)
**Metabolic function**			
Plasma glucose (mg/dl)	93.4 ± 1.9[b]<br>(68.8-127.6)	94.3 ± 2.3[b]<br>(65.8-139.5)	83.4 ± 2.2[a]<br>(56.5-139.9)
Serum insulin (µg/l)	0.84 ± 0.10[a]<br>(0.10-3.64)	0.59 ± 0.08[a]<br>(0.15-2.41)	0.87 ± 0.12[a]<br>(0.10-3.46)
Glucose to insulin ratio	270.0 ± 17.2[ab]<br>(65.0-739.4)	327.0 ± 21.5[b]<br>(61.4-843.5)	246.0 ± 20.9[a]<br>(53.7-756.9)
Serum fructosamine (mM)	1.39 ± 0.01[a]<br>(1.16-1.61)	1.41 ± 0.01[a]<br>(1.15-1.58)	1.44 ± 0.01[a]<br>(1.29-1.76)
Body condition score (1-5)	3.7 ± 0.1[a]<br>(2.0-5.0)	3.5 ± 0.1[a]<br>(2.0-5.0)	3.7 ± 0.1[a]<br>(2.0-5.0)

[a,b,c]Row values for each parameter differ significantly across the summer, rainy and winter seasons ($P < 0.05$).
