# Supplementary material for: Physiological changes in captive elephants in Northern Thailand as a result of the COVID-19 tourism ban – muscle, liver, metabolic function, and body condition
Source: Front Vet Sci. 2023 Dec 21;10:1303537. doi: 10.3389/fvets.2023.1303537 (PMC10764436; doi:10.3389/fvets.2023.1303537)
Supplement: Supplementary file 2 [file Data_Sheet_2.pdf]

Figures

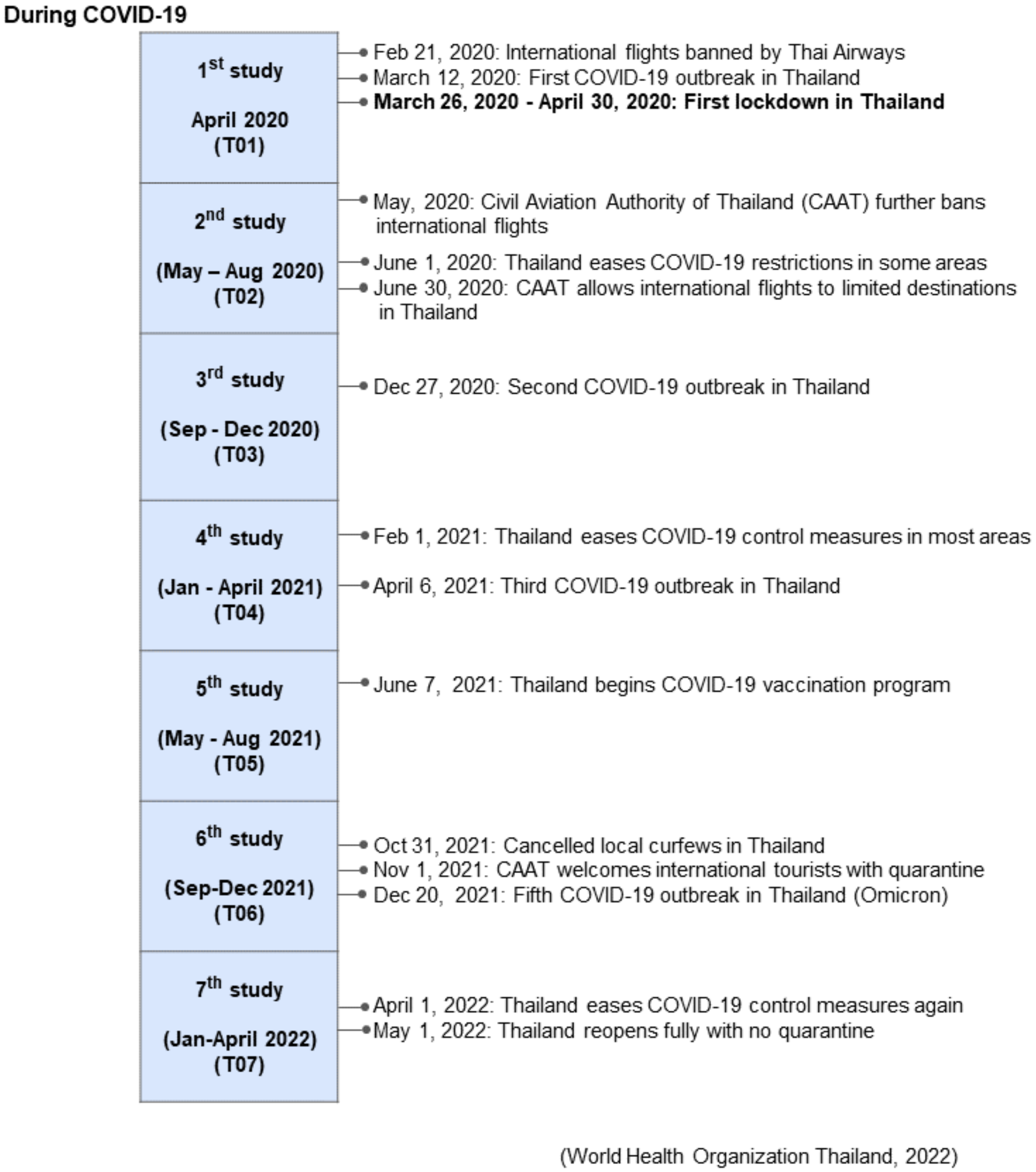

Figure 1. Associated events during the COVID-19 pandemic beginning in April 2020 (T01), and across seven periods of management surveys and blood sample collections (T02-T07) during the 2 years of the study from Supanta et al. (12).

A.

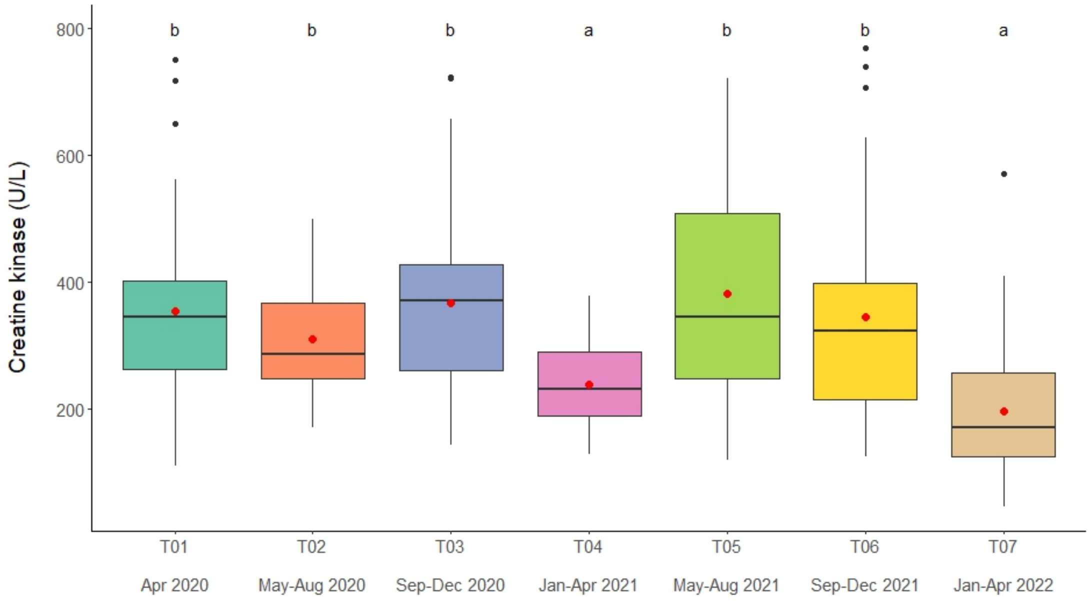
Creatine kinase (U/L)
800
600
400
200
b
b
b
a
b
b
a
T01
T02
T03
T04
T05
T06
T07
Apr 2020
May-Aug 2020
Sep-Dec 2020
Jan-Apr 2021
May-Aug 2021
Sep-Dec 2021
Jan-Apr 2022

B.

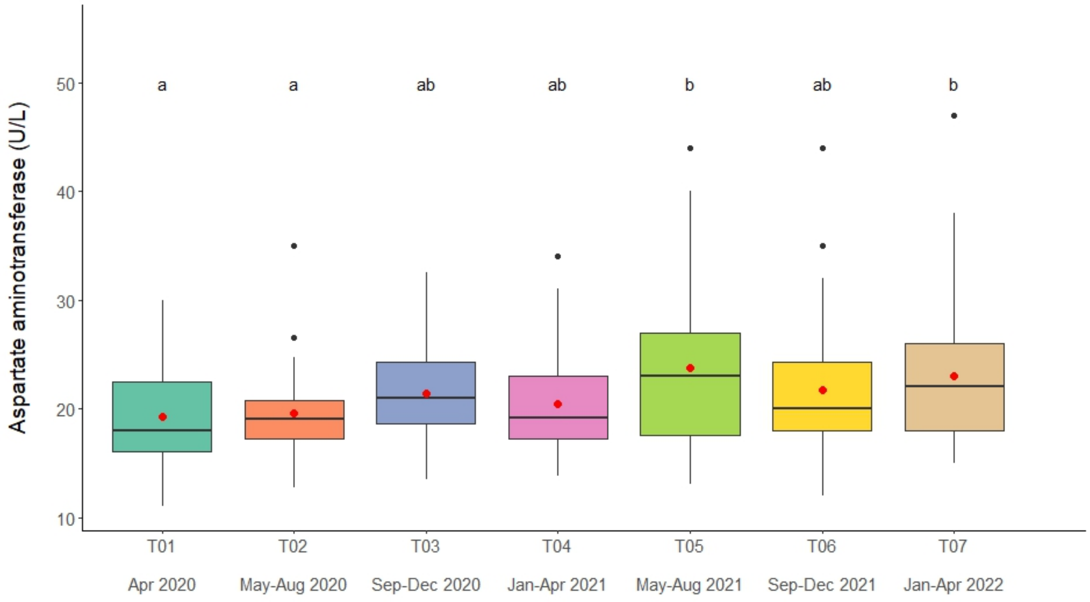
Aspartate aminotransferase (U/L)
50
40
30
20
10
a
a
ab
ab
b
ab
b
T01
T02
T03
T04
T05
T06
T07
Apr 2020
May-Aug 2020
Sep-Dec 2020
Jan-Apr 2021
May-Aug 2021
Sep-Dec 2021
Jan-Apr 2022

C.

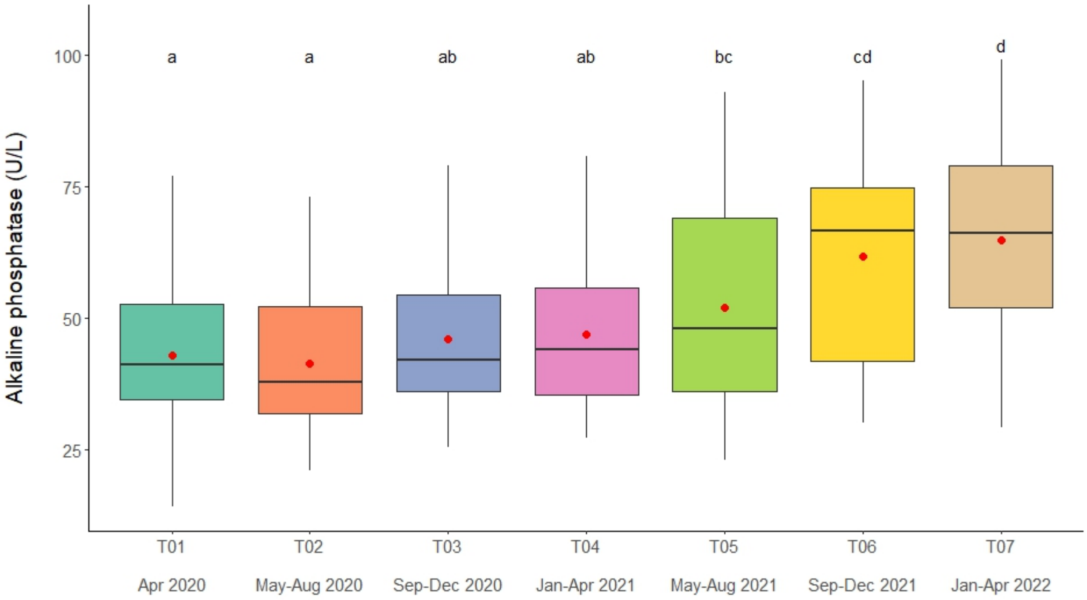

D.

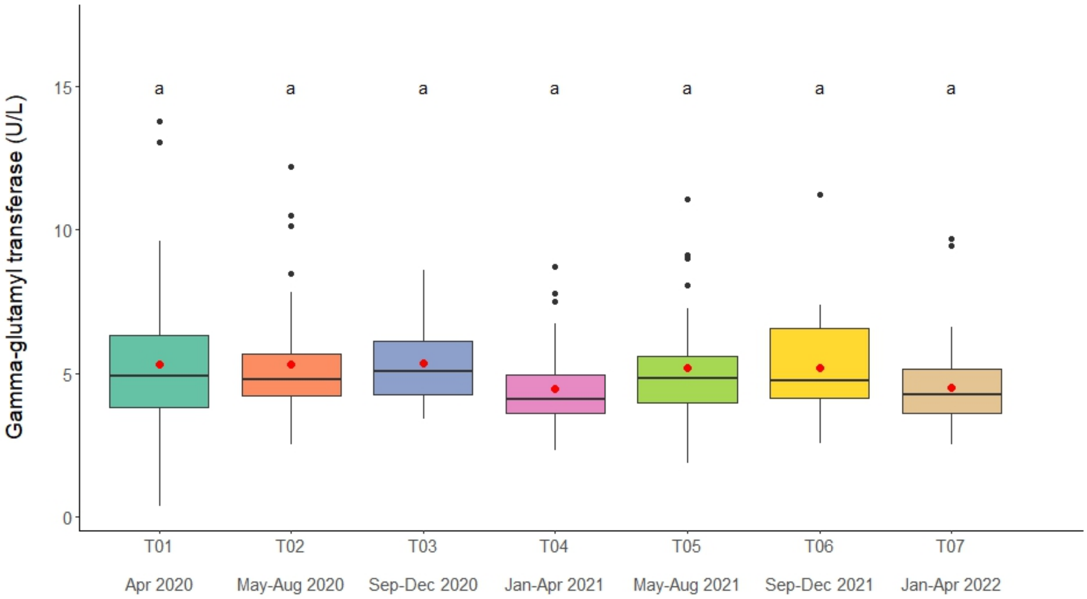

Figure 2. Boxplots of muscle and liver enzyme concentrations, (A) creatine kinase, (B) aspartate aminotransferase, (C) alkaline phosphatase, and (D) gamma-glutamyl transferase in captive Asian elephants (n= 58) over a 2-year period during the COVID-19 pandemic and international travel ban. Each plot shows the median (horizontal line), mean (red dot), 25% and 75% quartile ranges, and minimum and maximum ranges (whiskers). Different letters indicate significant differences between time periods ($P < 0.001$).

A.

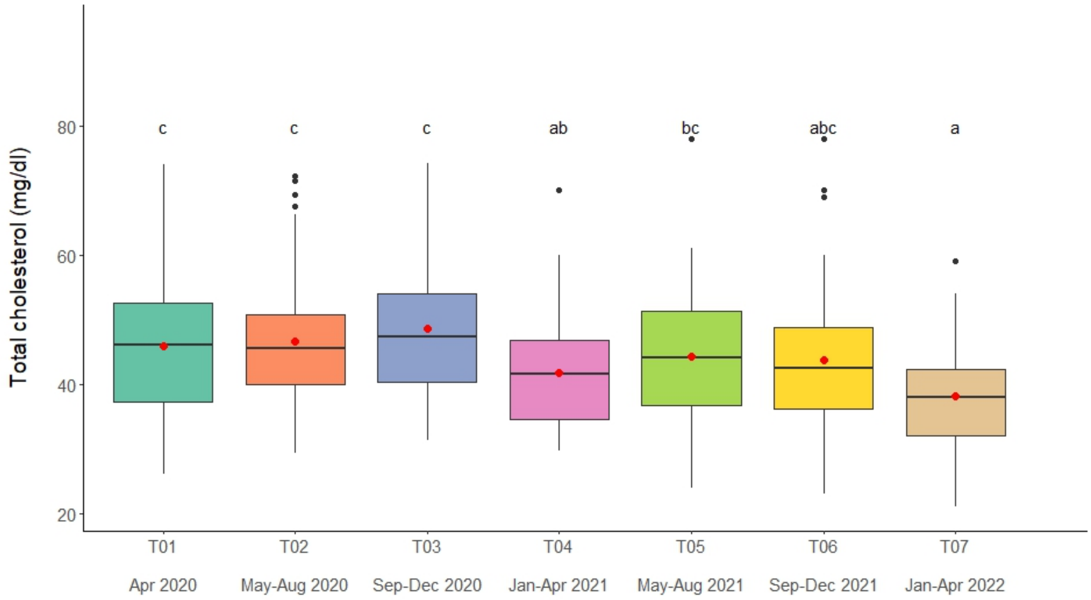
Total cholesterol (mg/dl)
80
60
40
20
c
c
c
ab
bc
abc
a
T01
T02
T03
T04
T05
T06
T07
Apr 2020
May-Aug 2020
Sep-Dec 2020
Jan-Apr 2021
May-Aug 2021
Sep-Dec 2021
Jan-Apr 2022

B.

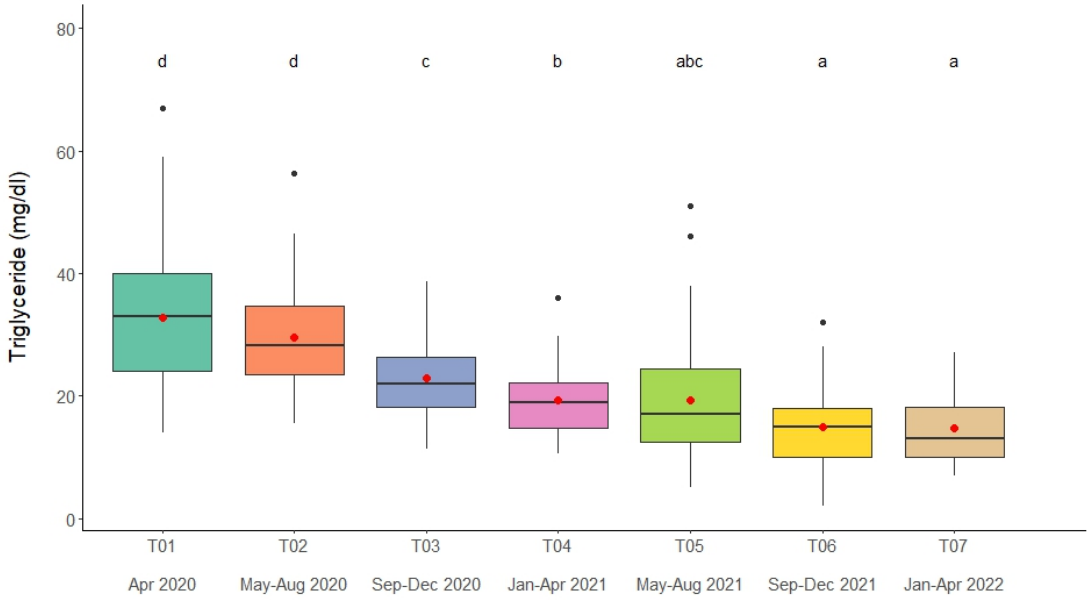
Triglyceride (mg/dl)
80
60
40
20
0
d
d
c
b
abc
a
a
T01
T02
T03
T04
T05
T06
T07
Apr 2020
May-Aug 2020
Sep-Dec 2020
Jan-Apr 2021
May-Aug 2021
Sep-Dec 2021
Jan-Apr 2022

C.

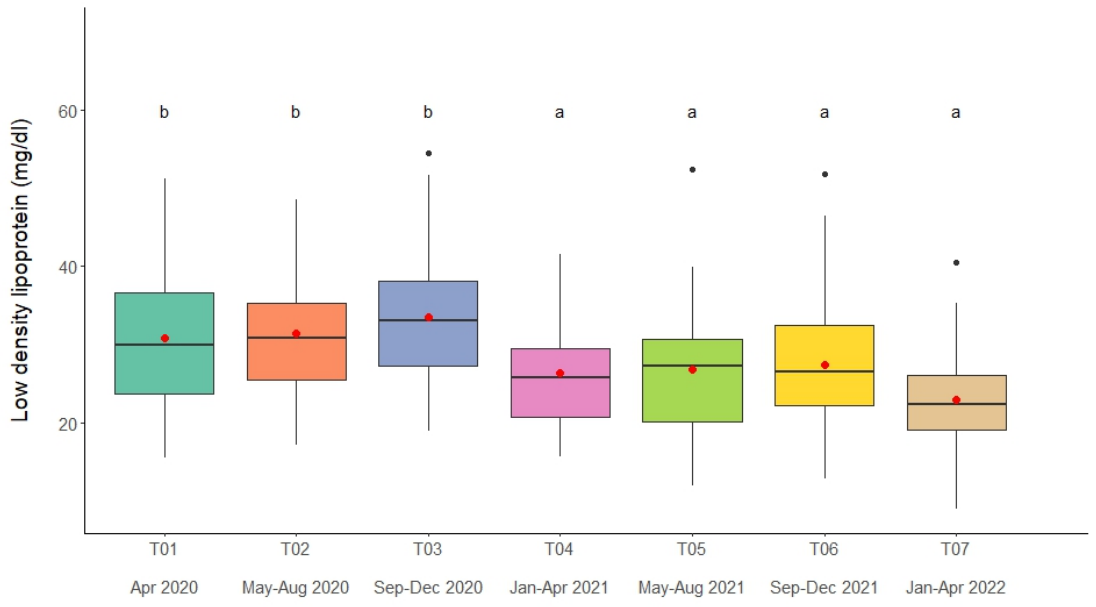

D.

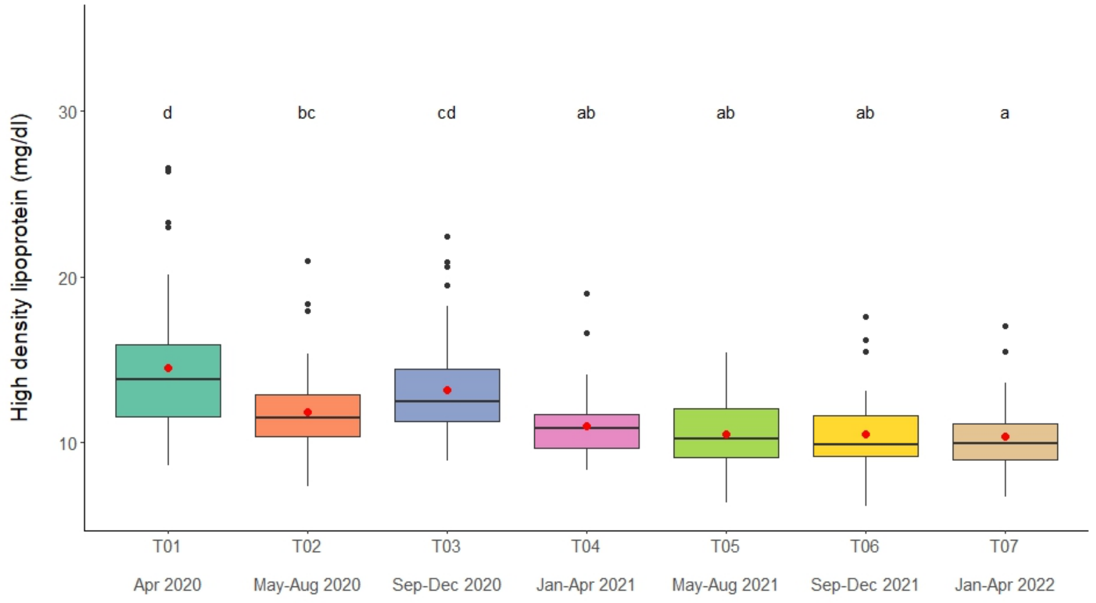

Figure 3. Boxplots of lipid profiles (A) total cholesterol, (B) triglycerides, (C) low density lipoproteins, and (D) high density lipoproteins in captive Asian elephants (n=58) over a 2-year period during the COVID-19 pandemic and international travel ban. Each plot shows the median (horizontal line), mean (red dot), 25% and 75% quartile ranges, and minimum and maximum ranges (whiskers). Different letters indicate significant differences between time periods (P <0.001).

A.

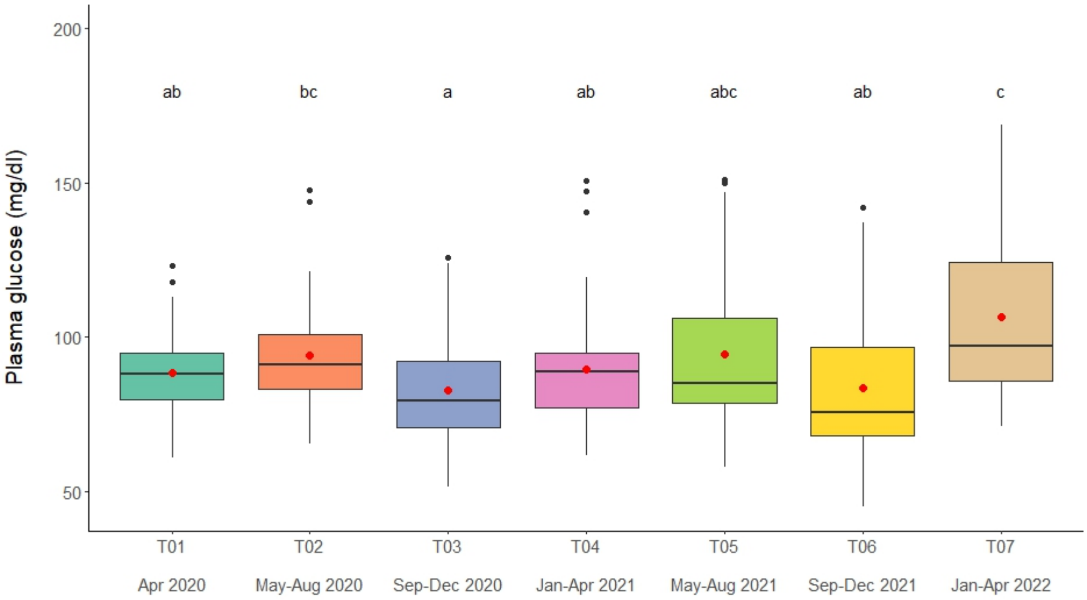
Plasma glucose (mg/dl)
200
150
100
50
ab
bc
a
ab
abc
ab
c
T01
Apr 2020
T02
May-Aug 2020
T03
Sep-Dec 2020
T04
Jan-Apr 2021
T05
May-Aug 2021
T06
Sep-Dec 2021
T07
Jan-Apr 2022

B.

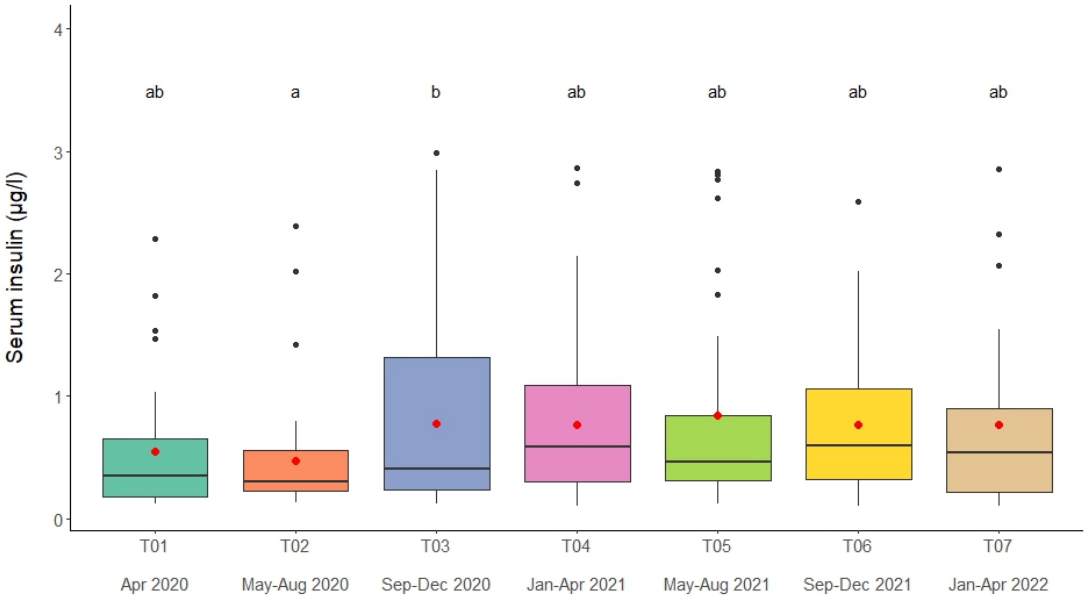
Serum insulin (µg/l)
4
3
2
1
0
ab
a
b
ab
ab
ab
ab
T01
Apr 2020
T02
May-Aug 2020
T03
Sep-Dec 2020
T04
Jan-Apr 2021
T05
May-Aug 2021
T06
Sep-Dec 2021
T07
Jan-Apr 2022

C.

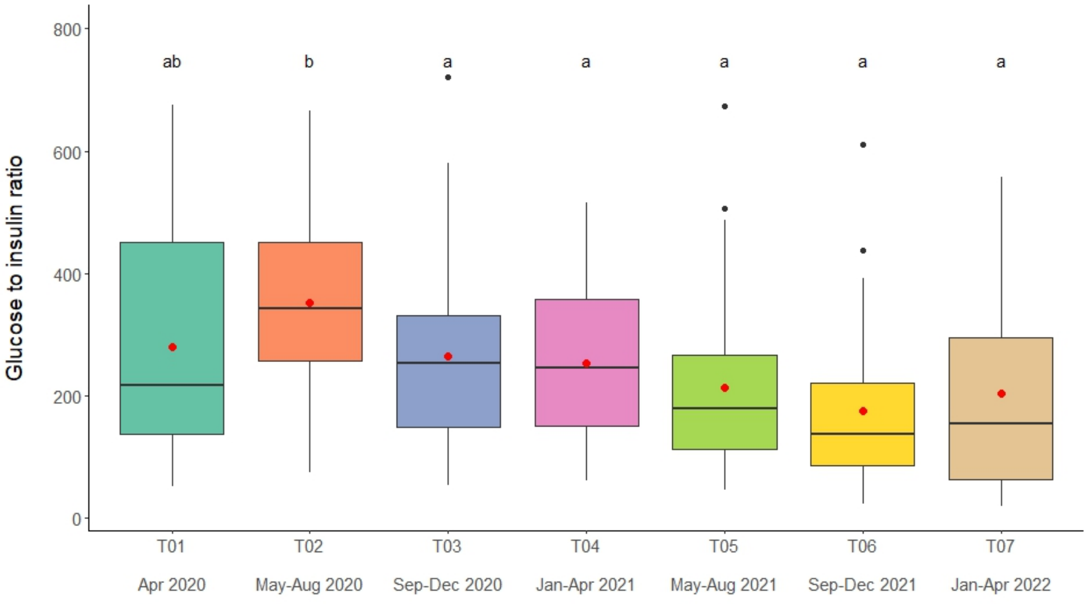
Glucose to insulin ratio
800
600
400
200
0
ab
b
a
a
a
a
a
T01
T02
T03
T04
T05
T06
T07
Apr 2020
May-Aug 2020
Sep-Dec 2020
Jan-Apr 2021
May-Aug 2021
Sep-Dec 2021
Jan-Apr 2022

D.

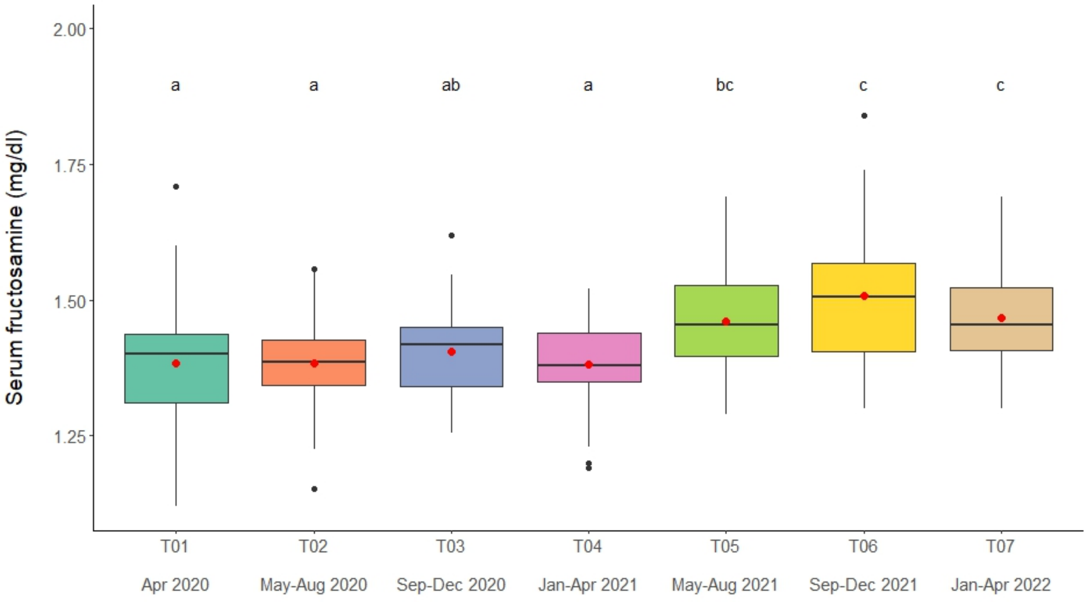
Serum fructosamine (mg/dl)
2.00
1.75
1.50
1.25
a
a
ab
a
bc
c
c
T01
T02
T03
T04
T05
T06
T07
Apr 2020
May-Aug 2020
Sep-Dec 2020
Jan-Apr 2021
May-Aug 2021
Sep-Dec 2021
Jan-Apr 2022

E.

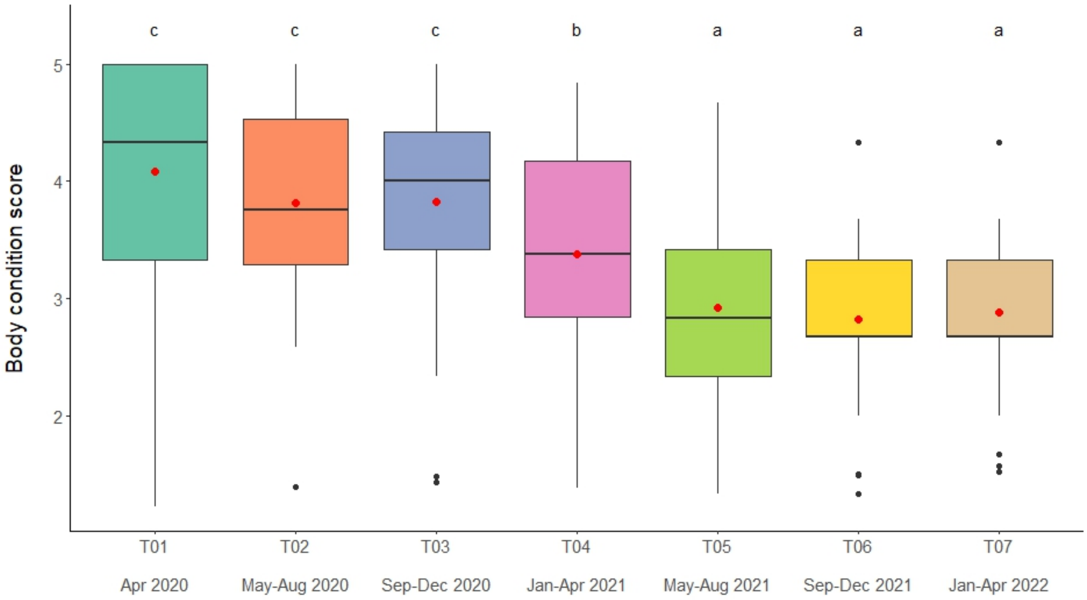

Figure 4. Boxplots of (A) plasma glucose, (B) serum insulin (C) glucose to insulin ratio (D) serum fructosamine and (E) body condition scores in captive Asian elephants (n=58) over a 2-year period during the COVID-19 pandemic and international travel ban. Each plot shows the median (horizontal line), mean (red dot), 25% and 75% quartile ranges, and minimum and maximum ranges (whiskers). Different letters indicate significant differences between time periods (P <0.001).
