# Supplementary material for: Physiological changes in captive elephants in Northern Thailand as a result of the COVID-19 tourism ban – muscle, liver, metabolic function, and body condition
Source: Front Vet Sci. 2023 Dec 21;10:1303537. doi: 10.3389/fvets.2023.1303537 (PMC10764436; doi:10.3389/fvets.2023.1303537)
Supplement: Supplementary file 4 [file Data_Sheet_4.docx]

**Mahout questionnaire**

**Time…….**

**Interviewer**......................................................................................................

**Date**..................................................................................................................

**Camp** **name**....................................................................................................

**1. Mahout information**

1.1 Name.........................................................................................................

1.2 Sex:  □ Male □ Female

1.3 Age...........................years old

1.4 Race...........................................................................................................

1.5 Nationality

1.6 Present address..........................................................................................

1.7 Economic status

Income:

□ Daily...........................................baht per day

□ Monthly......................................baht per month

Part-time:

□ No

□ Yes: please clarify ......................................................................

Income:

□ Daily...........................................baht per day

□ Monthly......................................baht per month

Total income per month.......................................baht

1.8 Cause of decreasing salary

……………………………………………………………………………

**2. Elephant information**

2.1 Elephant name:

□ Plai  □ Sridor  □ Pung.......................................................

2.2 Sex:  □ Male  □ Female

2.3 Age............................................................................................

2.4 BCS (Body condition score)......................................................

2.5 Weight…………………………………………………………

2.3 Behavior changing

□ No

□ Yes: please clarify………………………………………………………

□ Musth duration………………………………………………………

□ Heat duration………………………………………………………….

**3. Elephant working**

3.1 Elephant’s activity or work

□ No

□ Carry tourist

□ Trek

□ Show

□ Bathing

□ Others..................................................

3.2 Period of working

Time to start.................................................

Time to stop.................................................

3.3 Break time

□ No

□ Yes: please clarify ......................................................................

3.4 Restraint equipment

□ No

□ Yes: please clarify ......................................................................

**Type of restraint equipment**

□ Hook □ Nail □ Knife

□ Slingshot □ Chain □ Others.....................

When mahout use the restraint equipment?

………………………………………………………………………………

3.5 Trekking elephant

How many rounds per day? ............rounds

3.6 How many kilometers of the track per round? ........... kilometers

3.7 How long does it take for 1 round? .............................minutes

3.8 How much walking rate?.….. km/hr

3.9 Other activities?

□ No □ Yes: please clarify ........................................................................

3.10 Problem after work

□ No

□ Yes: please clarify ............................................................................

3.11 Cause of injury

□ Restraint equipment: please clarify................................................

□ Saddle equipment

□ Improper pathway

□ Elephant attack

□ Weakness from hard work

□ Others……………………………..…………………………………

3.12 Day off

□ No □ Yes: please clarify ....................................................................

**4. Rest area**

4.1 Do they chain elephant in rest area?

□ No □ Yes

Type of rest area

□ Forest: □ Chain □ Free

□ Field: □ Chain □ Free

□ Outdoor in camp: □ Chain  □ Free

□ Chain under tree

□ Chain in building

□ Corral: □ Chain □ Free

4.2 Number of elephants each type of rest area.........elephant per rest area

4.3 When do they chain elephant in rest area?

□ All the time □ During no work □ Nighttime

How long you chain elephant in rest area?.................hours

4.4 Chain

|  | Length of chain | Size of chain |
| --- | --- | --- |
| Male | .....................meters | ...........................units |
| Female | .....................meters | ...........................units |

**5. Nutrition**

5.1.1 Roughage and supplement food

□ Napier grass □ Banana tree □ Bana grass

□ Sugar cane □ Corn stalk □ Hey

□ Local roughage: please clarify...........................

| Amount/day | No. of feeding/ day | Amount/feeding |
| --- | --- | --- |
|  |  |  |

5.1.2 Concentrate food

□ No

□ Yes: □ Pellet food elephant (% protein) .........

□ Others...............................................................................

| Amount/day | No. of feeding/ day | Amount/feeding |
| --- | --- | --- |
|  |  |  |

5.1.3 Others

□ No

□ Yes: □ Tablet vitamin □ Herb

□ Sticky rice □ Tamarind

□ Others......................................................................................

| Amount/day | No. of feeding/ day | Amount/feeding |
| --- | --- | --- |
|  |  |  |
