# Supplementary figures and images for: Physiological changes in captive elephants in Northern Thailand as a result of the COVID-19 tourism ban – muscle, liver, metabolic function, and body condition
Source: Front Vet Sci. 2023 Dec 21;10:1303537. doi: 10.3389/fvets.2023.1303537 (PMC10764436; doi:10.3389/fvets.2023.1303537)

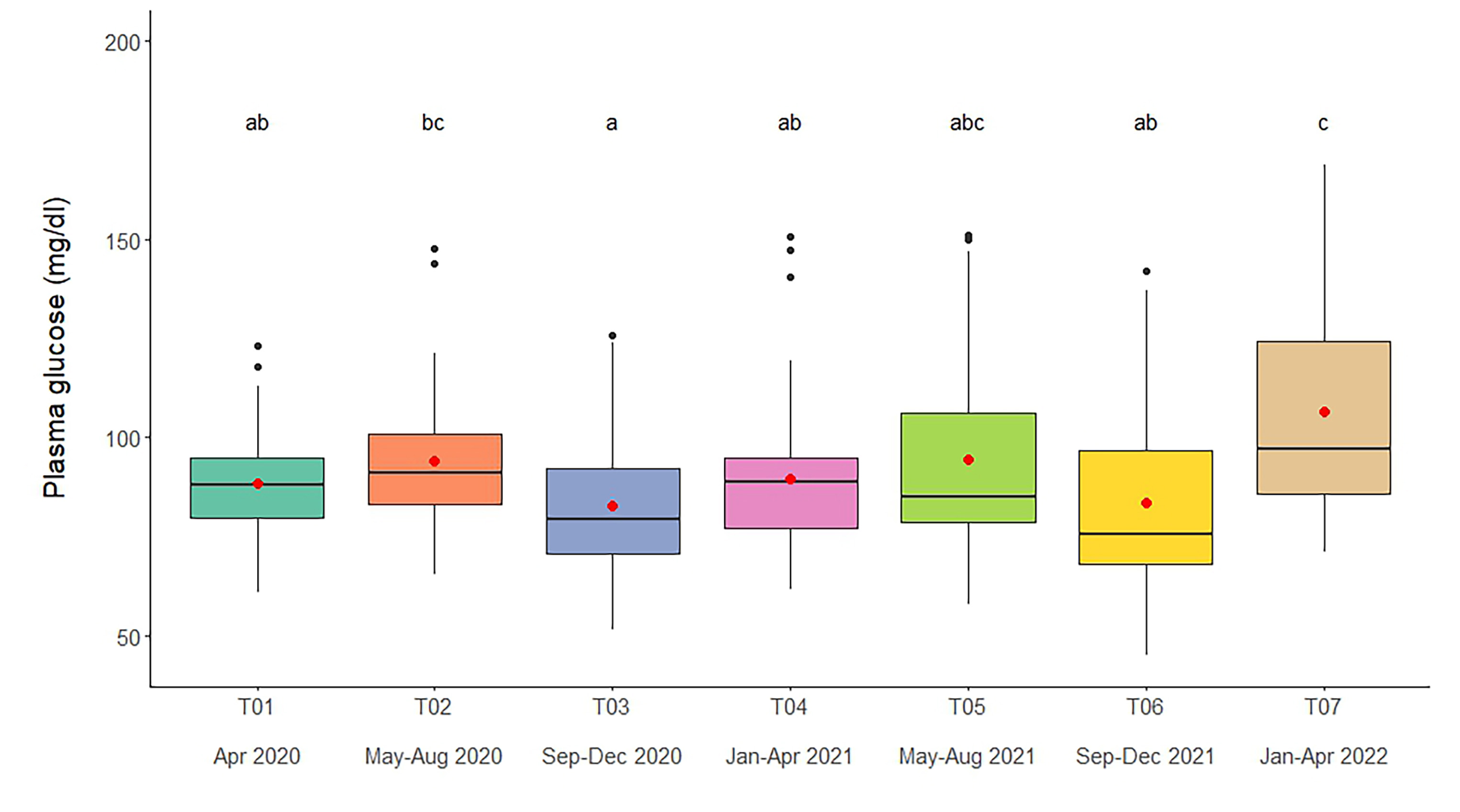

Supplement: Supplementary file 5 [file Image_1.JPEG]

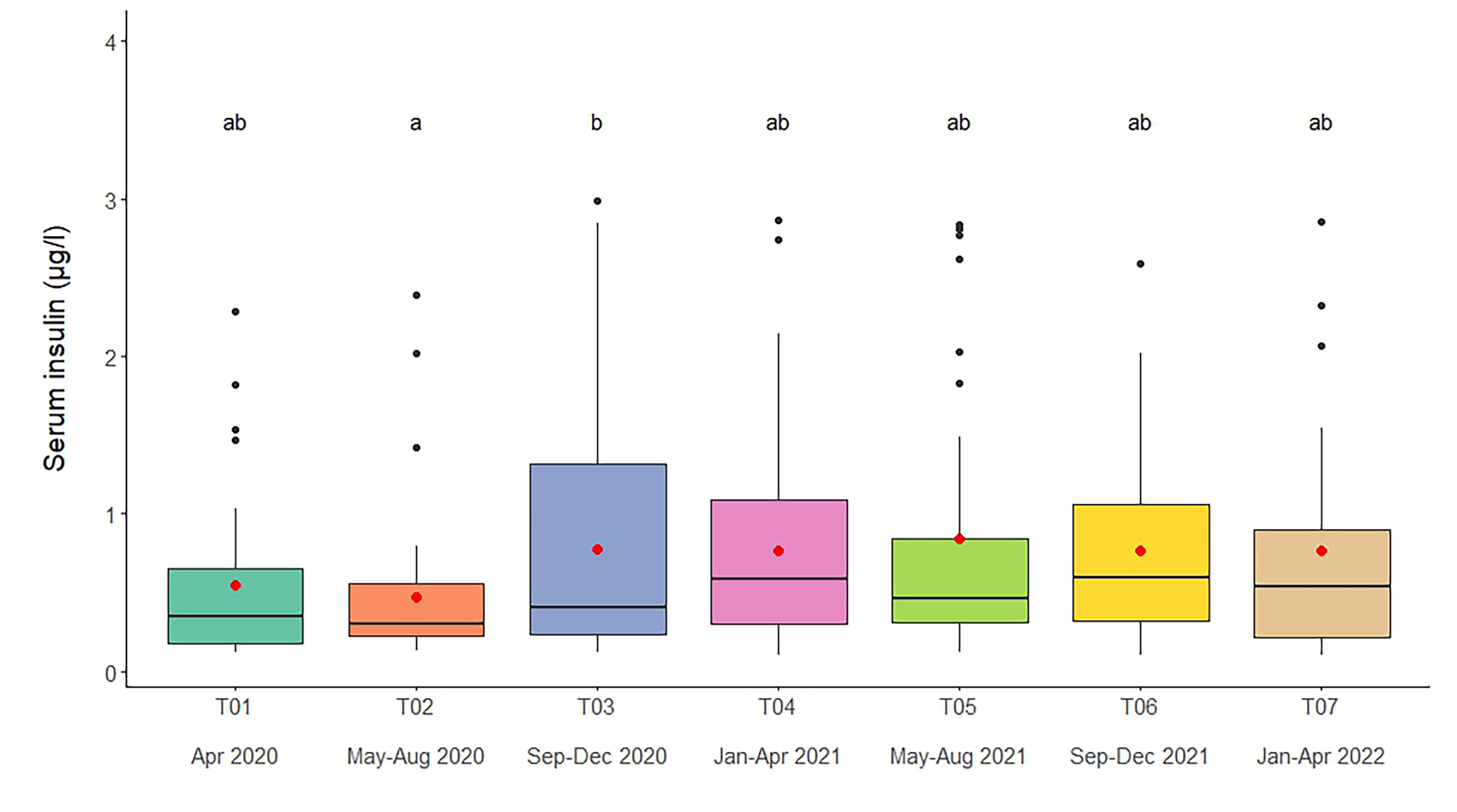

Supplement: Supplementary file 6 [file Image_2.JPEG]

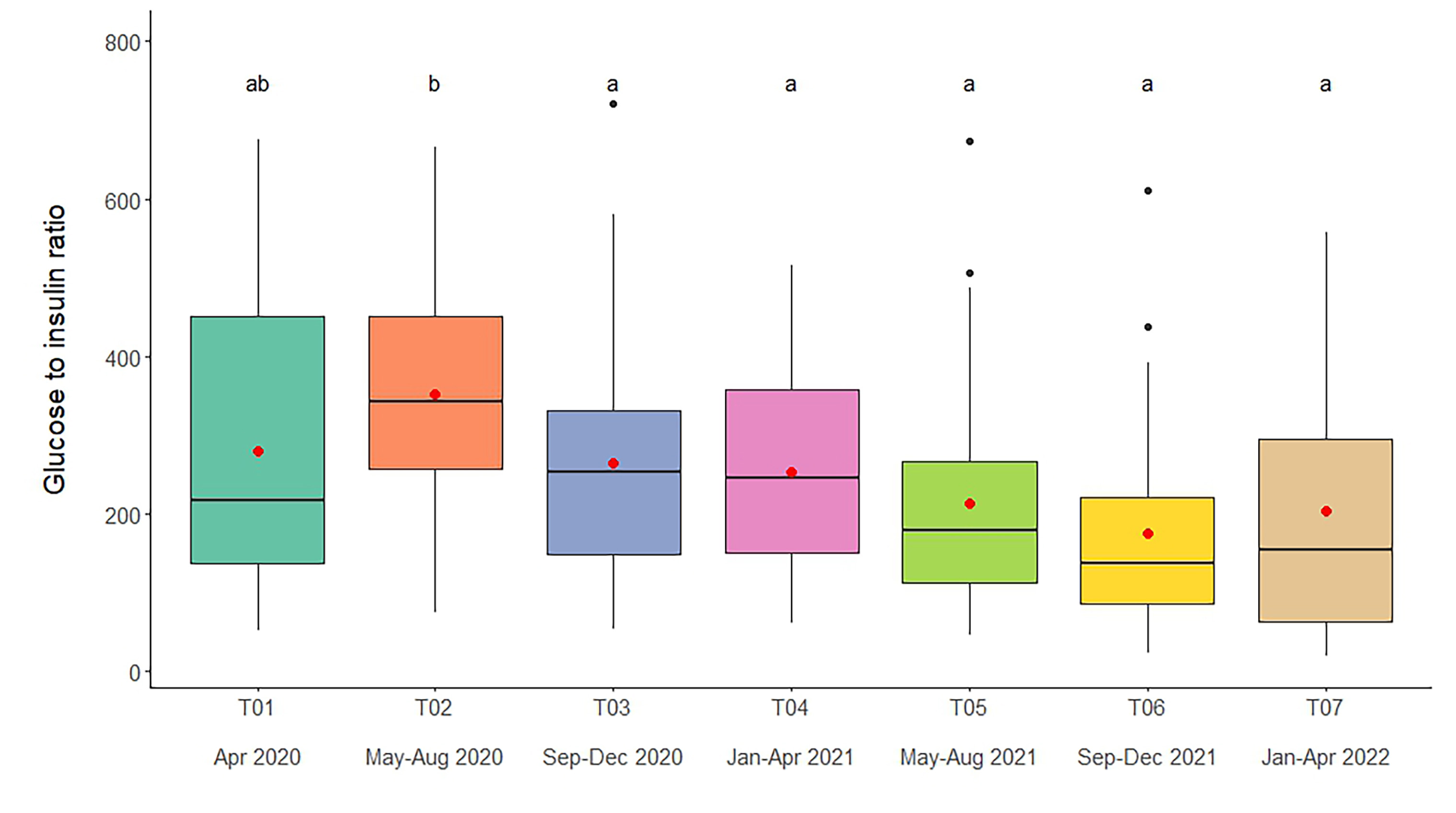

Supplement: Supplementary file 7 [file Image_3.JPEG]

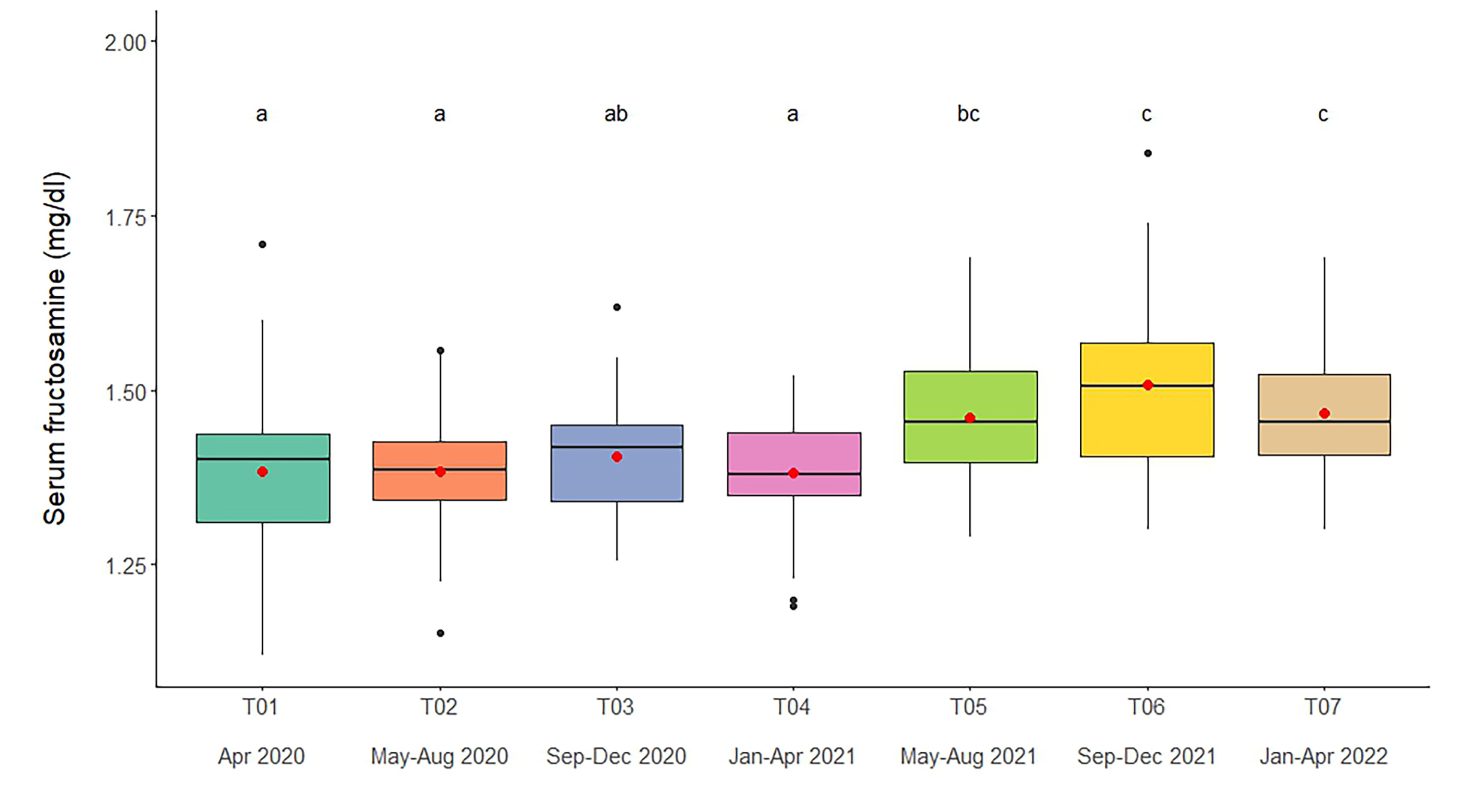

Supplement: Supplementary file 8 [file Image_4.JPEG]
